# Cowpea mosaic virus intratumoral immunotherapy maintains stability and efficacy after long‐term storage

**DOI:** 10.1002/btm2.10693

**Published:** 2024-07-07

**Authors:** Andrea Simms, Zhongchao Zhao, Edward Cedrone, Marina A. Dobrovolskaia, Nicole F. Steinmetz

**Affiliations:** ^1^ Aiiso Yufeng Li Family Department of Chemical and Nano Engineering University of California, San Diego La Jolla California USA; ^2^ Center for Nano‐ImmunoEngineering University of California, San Diego La Jolla California USA; ^3^ Moores Cancer Center University of California, San Diego La Jolla California USA; ^4^ Shu and K.C. Chien and Peter Farrell Collaboratory University of California La Jolla California USA; ^5^ Nanotechnology Characterization Laboratory, Cancer Research Technology Program Frederick National Laboratory for Cancer Research sponsored by the National Cancer Institute Frederick Maryland USA; ^6^ Department of Bioengineering University of California, San Diego La Jolla California USA; ^7^ Department of Radiology University of California, San Diego La Jolla California USA; ^8^ Institute for Materials Discovery and Design University of California La Jolla California USA; ^9^ Center for Engineering in Cancer, Institute for Engineering in Medicine University of California La Jolla California USA

**Keywords:** cowpea mosaic virus, intratumoral immunotherapy, long‐term stability, melanoma, translational drug development

## Abstract

Cowpea mosaic virus (CPMV) has demonstrated superior immune stimulation and efficacy as an intratumoral immunotherapy, providing a strong argument for its clinical translation. One important consideration for any new drug candidate is the long‐term stability of the drug and its formulation. Therefore, our lab has evaluated the physical stability and biological activity, that is, anti‐tumor potency, of formulations of CPMV in buffer (with and without a sucrose preservative) in multiple temperature conditions ranging from ultralow freezers to a heated incubator over a period of 9 months. We found that non‐refrigerated temperatures 37°C and room temperature quickly led to CPMV destabilization, as evidenced by significant protein and RNA degradation after just 1 week. Refrigerated storage at 4°C extended physical stability, though signs of particle breakage and RNA escape appeared after 6 and 9 months. CPMV stored in frozen conditions, including −20°C, −80°C, and liquid N_2_, remained intact and matched the characteristics of fresh CPMV throughout the duration of the study. The biological activity was evaluated using a murine dermal melanoma model, and efficacy followed the observed trends in physical stability: CPMV stored in refrigerated and warmer conditions exhibited decreased anti‐tumor efficacy compared to freshly prepared formulations. Meanwhile, frozen‐stored CPMV performed similarly to freshly purified CPMV, resulting in reduced tumor growth and extended survival. Data, therefore, indicates that CPMV stored long‐term in cold or frozen conditions remains stable and efficacious, providing additional support to advance this powerful plant virus to translation.

**Translational Impact Statement**

Cowpea mosaic virus (CPMV) is a potent adjuvant for tumor‐ and tissue‐agnostic intratumoral immunotherapy. Foundational work delineates the mechanism of action of CPMV to modulate the tumor microenvironment. Toward translating this novel anti‐cancer drug candidate toward the clinic, this study investigates the longitudinal and thermal stability of aqueous formulations of CPMV and concludes the best long‐term storage condition is −20°C. This work provides extensive stability‐indicating data that further supports the push for clinical translation of CPMV.

## 1 INTRODUCTION

Cowpea mosaic virus (CPMV) is being developed as a drug candidate for intratumoral immunotherapy with demonstrated anti‐tumor efficacy in multiple murine tumor models, such as melanoma,^1^, ^2^, ^3^ intracranial glioma,^4^ and ovarian,^1^, ^5^, ^6^ colon,^1^ and breast^1^ cancers. When compared to other plant viruses and virus‐like particles, including those from the same family,^2^ of different size/shape,^7^ of the same icosahedral shape,^3^ or even the same virus but devoid of the genomic RNA,^8^ wild‐type CPMV consistently demonstrates superior anti‐tumor efficacy. Following success with mouse cancer models, CPMV intratumoral immunotherapy has shown treatment success against canine oral melanoma,^9^, ^10^ invasive squamous cell carcinoma,^10^ and canine inflammatory mammary cancer.^11^

CPMV is an icosahedral plant virus of the genus *Comovirus*.^12^ It has a diameter of ~30 nm with a pseudo‐T = 3 symmetry and is composed of 60 identical copies of a large (*L* = 42 kDa) and small (*S* = 24 kDa) coat protein (CP). The bipartite, positive‐sense, single‐stranded RNAs (RNA‐1, 6.0 knts and RNA‐2, 3.5 knts) are encapsidated into isometric particles.^12^ While CPMV is named for its infection of cowpea plants, it can also infect other legumes and has been propagated experimentally in *Nicotiana benthamiana*.^13^ It has long been stated that CPMV does not replicate in human cells, and recent data from our lab corroborates this claim (Omole et al., will be reported elsewhere).

Unlike oncolytic viruses, CPMV does not replicate in mammalian cells or lyse cancer cells directly—rather, CPMV is immunomodulatory and modulates an immunosuppressive tumor microenvironment (TME) from immunologically cold to hot, thus priming potent anti‐tumor immunity through several mechanisms: CPMV alerts the immune system by presenting pathogen‐associated molecular patterns (PAMPs) to activate toll‐like receptors (TLRs). The capsid proteins are recognized by TLRs 2 and 4 and their RNA by TLR 7 to induce inflammatory cytokines and chemokines such as type I interferon (IFN‐β) and interferon‐gamma (IFN‐γ), IL‐12, and granulocyte‐macrophage colony‐stimulating factor (GM‐CSF).^14^ CPMV is more than just a TLR agonist, though. The highly organized and repetitive nature of the viral capsid assembly itself can be viewed as PAMPs; immunostimulation is also provided through engagement with complement and presentation of Thelper epitopes.^15^ CPMV acts on innate immune cells to activate and recruit more innate immune cells into the TME, and it is the innate immune cells (M1 macrophages, N1 neutrophils) as well as natural killer (NK) cells that induce tumor cell death and subsequently process tumor‐associated antigens and neoantigens. Systemic immunity (abscopal effect) was demonstrated in two‐tumor models and re‐challenge studies.^16^

Given the demonstrated potency of CPMV and the understanding of its mechanism of action, we are now pursuing the translational development of this drug candidate. Translational challenges of biologics drug candidates involve the product's shelf life and potential cold chain complications. Proteins tend to be sensitive to stresses such as thermal changes, photo exposure, and mechanical agitation, which may trigger degradation and decrease the efficacy and safety of the product.^17^ Physical instability of proteins often manifests in the form of aggregation during long‐term storage.^17^, ^18^, ^19^, ^20^ Aggregation primarily occurs because it is thermodynamically more favorable for partially denatured protein chains to interact with each other than for their exposed hydrophobic residues to interact with the aqueous solvent.^19^ Physically unstable proteins may have modified activity, immunogenicity, and solubility, which can make the product more dangerous than useful. One example of protein aggregates becoming dangerous is the formation of amyloid fibrils, which is associated with multiple neurodegenerative diseases.^18^ It is therefore important to evaluate the conditions in which CPMV becomes unstable or inactive and determine the best long‐term storage condition(s).

A previous study from our lab found that although plant viruses are considered stable, CPMV showed signs of structural instability and degradation after 1 month at 25 and 37°C.^21^ Therefore, in the present study, we evaluated CPMV stability and biological efficacy in a longitudinal study upon storage at 37°C, room temperature (RT) shelf, 4°C refrigerator, −20°C as well as −80°C freezer, and liquid nitrogen (N_2_). CPMV was formulated in 0.1 M potassium phosphate (KP) buffer pH 7.0 with and without the addition of 20% sucrose, a preservative commonly used in the biopharmaceutical industry.^22^ Following storage after 1 week, 1 month, 3 months, 6 months, and 9 months, the physical integrity and biological activity were assayed.

## 2 MATERIALS AND METHODS

### 2.1 Production of CPMV and long‐term storage conditions

CPMV nanoparticles were propagated in and purified from black‐eyed pea no. 5 plants with modifications to a previously described protocol (the detailed method is described in Supporting Information S1).^23^ Purified CPMV batches were combined, then filtered through a 0.22 μm syringe filter with 33 mm diameter (MilliporeSigma, St. Louis, MO, USA) and separated with one part filtered through a PD‐10 desalting column (Cytiva, Marlborough, MA, USA) pre‐conditioned with 0.1 M KP buffer pH 7.0 without sucrose and the other with 20% (w/v) sucrose added. This step was to both perform a final purification to remove any aggregates and to perform a buffer exchange to the final CPMV storage buffers. The initial concentrations of the two portions of CPMV were measured via ultraviolet–visible spectroscopy (UV–Vis, NanoDrop 2000, ThermoFisher Scientific, Waltham, MA, USA) using the CPMV specific extinction coefficient (*ε*) of 8.1 mL/mg cm at 260 nm, then diluted to 10 mg/mL in their respective buffers.

### 2.2 CPMV storage and characterization

Aliquots of 50 μL of 10 mg/mL CPMV in each buffer were placed in polypropylene tubes and separated between six different temperature conditions: 37°C incubator, RT, 4°C refrigerator, −20°C freezer, −80°C freezer, and liquid N_2_ dewar. Ten milligrams per milliliter was used because we propose this concentration for vialing for future clinical trials, and historically, at this concentration, CPMV is stable and sufficiently concentrated for intratumoral injection. Fifty microliter aliquots were sufficient to complete all stability‐indicating assays at each time point. CPMV particles stored in the different conditions were characterized at 7 days, 1 month, 3 months, 6 months, and 9 months (9 months excluding 37°C and RT samples). For each time point, samples were compared to freshly purified CPMV. For characterization at each testing time point, two aliquots of CPMV in 0.1 M KP buffer pH 7.0 with and without sucrose were removed and diluted with 450 μL of 0.1 M KP buffer pH 7.0. Samples were stored at 4°C between tests. At 3 months of storage from a separate CPMV preparation, aliquots of CPMV stored at −20°C were subjected to 10 thaw–freeze cycles of 24 h thawing at RT and 24 h freezing at −20°C.

### 2.3 Endotoxin quantification

The threshold pyrogen dose of endotoxin recognized by the U.S. FDA is 5.0 EU/kg for all administration routes but intrathecal.^24^ Based on an estimated CPMV efficacious dose for humans of 10 mg/tumor, assuming a human body weight of 70 kg, and using the dose conversion between mice and humans guide found in the FDA guidance for industry,^25^ the endotoxin limit for CPMV was determined as 35.7 EU/mg. Endotoxin levels in CPMV preparations were quantified using a Pierce Chromogenic Endotoxin Quant Kit (ThermoFisher Scientific, Waltham, MA, USA). If the endotoxin load was over the limit, a previously published protocol using Triton X‐114 was used to remove endotoxins.^8^

### 2.4 Physicochemical characterization

CPMV structural stability was analyzed via UV–Vis spectroscopy, sodium dodecyl sulfate‐polyacrylamide gel electrophoresis (SDS‐PAGE), native agarose gel electrophoresis, bioanalyzer, size exclusion chromatography, dynamic light scattering (DLS), transmission electron microscopy (TEM), and liquid chromatography with tandem mass spectrometry (LC–MS/MS). Detailed procedures for each of these methods are given in Supporting Information S1.

### 2.5 B16F10 tumor inoculation and intratumoral treatment

B16F10 cells (ATCC CRL‐6475, Manassas, VA, USA) were cultured using Dulbecco's Modified Eagle's Medium with 4.5 g/L glucose and L‐glutamine (Corning, Glendale, AZ, USA), supplemented with 10% (v/v) heat‐inactivated fetal bovine serum (R&D Systems, Minneapolis, MN, USA) and 1% (v/v) penicillin–streptomycin (Cytiva, Marlborough, MA, USA). Cells were grown in an incubator at 37°C and 5% CO_2_. For harvesting, cells were first washed twice with phosphate‐buffered saline (PBS), then removed from flasks using 0.05% trypsin–EDTA (Corning, Glendale, AZ, USA). B16F10 cells were then resuspended in sterile PBS at 6.67 × 10^6^ cells/mL.

Six‐week‐old male or female mice were purchased from The Jackson Laboratory (Bar Harbor, ME, USA) and housed at the Moores Cancer Center at the University of California, San Diego (UCSD). The animals were given unlimited access to food and water, and all mouse studies were carried out in accordance with the guidelines of the Institutional Animal Care and Use Committee of UCSD under protocol S18021.

Two hundred thousand cells in 30 μL were injected intradermally into the shaved right flank of each mouse on Day 0. Tumor growth was monitored via mouse weight and caliper measurements (formula *l* × (*w* ^2^/2) was used to calculate volume). CPMV treatments began on Day 7 or 8 when tumors reached a minimum volume of 20 mm^3^. CPMV was injected intratumorally, 100 μg in 20 μL PBS, every 4 days after the initial treatment for a total of three injections. To assess and mitigate potential pain caused by tumors, all mice were carefully monitored for changes in appearance and behaviors, such as regression in mobility, hunched posture, rough hair, changes in respiration, or aggressive behavior. Mice were euthanized when their tumors reached 1000 mm^3^, if they lost >20% of their pre‐tumor inoculation weight, or if they showed signs of pain and discomfort as listed above. The number of animals used for each experiment are as follows:

1. Six months: *n* = 5 per group, 8 groups, total = 40 male mice.
2. Nine months: *n* = 5 per group, 6 groups, total = 30 male mice.
3. Three months follow‐up: *n* = 5 per group, 5 groups, total = 25 male mice. *N* = 4 reported for 4°C; one mouse was removed from the study and euthanized early on for non‐tumor‐related skin lesions, as recommended by the attending veterinarian.
4. Six months follow‐up: *n* = 5 per group, 4 groups per sex, total = 20 male mice and 20 female mice.

### 2.6 Cytokine assay

#### 2.6.1 Human donor blood

Human blood was collected from healthy donor volunteers under the institutional review board (IRB)‐approved NCI‐Frederick Protocol OH99‐C‐N046. The blood was drawn into vacutainers containing lithium heparin as an anticoagulant (BD, Franklin Lanes, NJ, USA) and processed within 2 h from the donation.

#### 2.6.2 Peripheral blood mononuclear cells isolation and cytokine secretion assay

Experiments were performed according to the NCL assay cascade protocols ITA‐10 (https://www.cancer.gov/nano/research/ncl/protocols-capabilities/ncl-method-ita-10.pdf) and ITA 27 (https://www.cancer.gov/nano/research/ncl/protocols-capabilities/ncl-method-ita-27.pdf), which also provide detailed information about reagent sources and concentrations. Briefly, whole blood anticoagulated with lithium heparin was diluted with PBC and used to purify peripheral blood mononuclear cells (PBMC) using the Ficoll Paque density gradient centrifugation procedure. PBS and agonists with known immunostimulatory properties (ODN2216 at 5 μg/mL and phytohemagglutinin, M form [PHA‐M] at 10 μg/mL) were used as the negative and positive controls (NC and PCs), respectively. PBMC from three healthy donor volunteers were incubated with controls and nanoparticles for 24 h. At the end of the incubation, the samples were centrifuged for 5 min at 18,000 *g*, and the supernatants were analyzed for the presence of cytokines and IFNs using custom multiplex enzyme‐linked immunosorbent assay (ELISA) kits (Quansys, Logan, UT, USA). The data were acquired using Q‐View software (Quansys, Logan, UT, USA) and analyzed using GraphPad Prism (Boston, MA, USA).

## 3 RESULTS AND DISCUSSION

**FIGURE 1:**
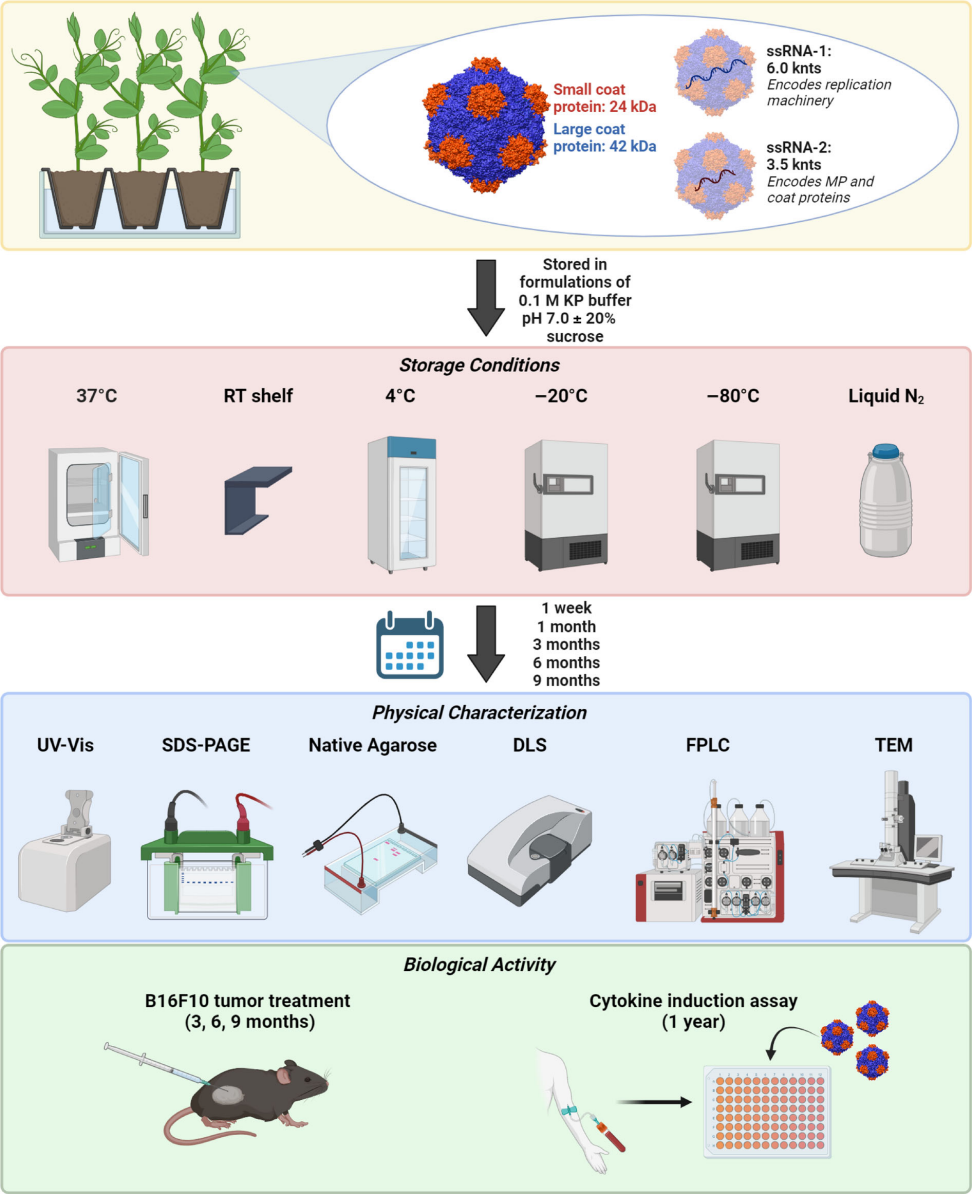
Cowpea mosaic virus (CPMV) long‐term stability experimental set‐up. CPMV was purified from infected black‐eyed pea no. 5 plants, then aliquoted and stored in six different temperature conditions: 37°C incubator, room temperature (RT) shelf, 4°C refrigerator, −20°C freezer, −80°C freezer, and liquid nitrogen (N_2_) dewar. After storage for 1 week, 1 month, 3 months, 6 months, and 9 months, the CPMV particles were characterized for structural stability via ultraviolet–visible spectroscopy (UV–Vis), agarose gel electrophoresis, sodium dodecyl sulfate‐polyacrylamide gel electrophoresis (SDS‐PAGE), dynamic light scattering (DLS), fast protein liquid chromatography (FPLC), and transmission electron microscopy (TEM). CPMV structure was created using UCSF Chimera version 1.16 using the Protein Data Bank entry 1ny7. Figure made in BioRender.com.

We evaluated the structural integrity and biological activity of CPMV after storage at 37°C, RT, 4°C, −20°C, −80°C, and liquid N_2_. We first obtained a baseline for how freshly prepared CPMV presents and, by extension, how CPMV should behave after long‐term storage. Figure 1 provides an outline of the experimental set‐up. This study was designed with the aid of chemistry, manufacturing, and controls (CMC) consultants as a prelude toward a larger formulation study as part of the CMC development for future clinical trials. For brevity, the data presented in the main text focuses on characterizations after 3 and 6 months storage. Additional time points can be found in Supporting Information S1.

### 3.1 Longitudinal CPMV physical stability in multiple storage conditions

We used UV–Vis spectroscopy as a first test to gain insights into the stability of CPMV upon storage (Figures S1 and S2). Previous data indicate that intact and pure CPMV is characterized by an *A*260/280 nm ratio of 1.7–1.8. While this range is widely stated in the literature, we note that we could not identify the original works that established this. Historically, we have used the *A*260/280 ratio as an indicator, yet not an absolute measure, of whether a CPMV preparation is intact and pure or not. In this study, we observed that overall, freshly prepared CPMV and samples stored refrigerated and colder maintained *A*260/280 nm ratios within the acceptable range of 1.7–1.8 over the time course, indicating stable formulations. The only samples that showed differences—albeit subtle—were samples stored at RT or 37°C. This may indicate some trend of degradation or aggregation, but it may also highlight the limitations of this simple read‐out to detect subtle structural changes. Of note, though samples stored at 37°C and RT were closed in tubes, the seals of the containers were evidently not tight, as there was visible evaporation from these samples, causing the observed concentration to increase over time.

**FIGURE 2:**
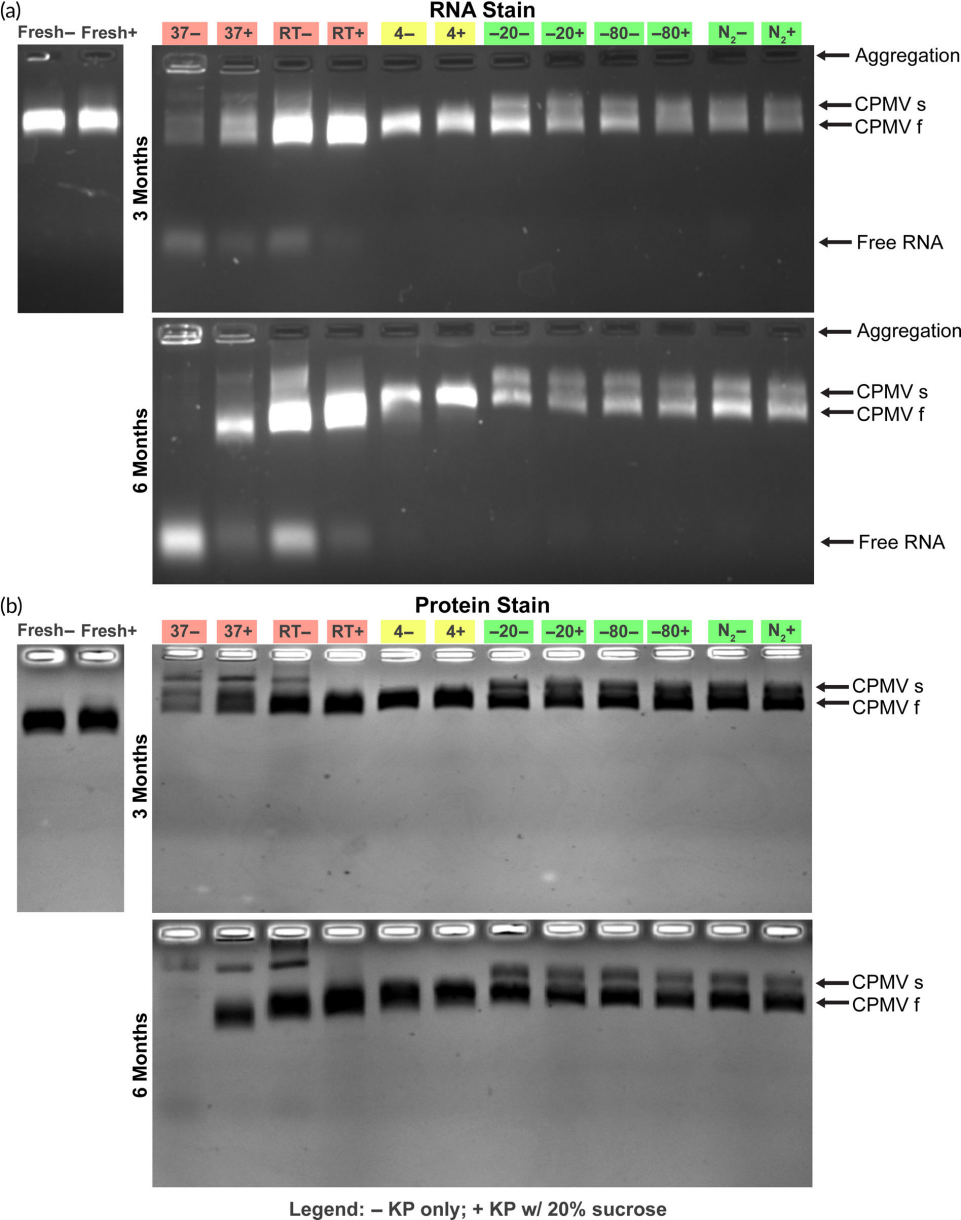
Native agarose gel electrophoresis. Cowpea mosaic virus (CPMV) particles were (a) stained with GelRed nucleic acid stain and imaged under ultraviolet light, and (b) stained with Coomassie Brilliant Blue and imaged under white light. A gel of fresh CPMV (leftmost) is compared to particles stored for 3 months (top) and 6 months (bottom). Additional time points can be found in Figures S1 and S2. Red highlighting identifies bands showing broken, aggregated, and/or RNA‐less CPMV; yellow indicates questionable bands in which CPMV appears completely cleaved but otherwise not broken or aggregated; and green indicates acceptable band patterns.

**FIGURE 3:**
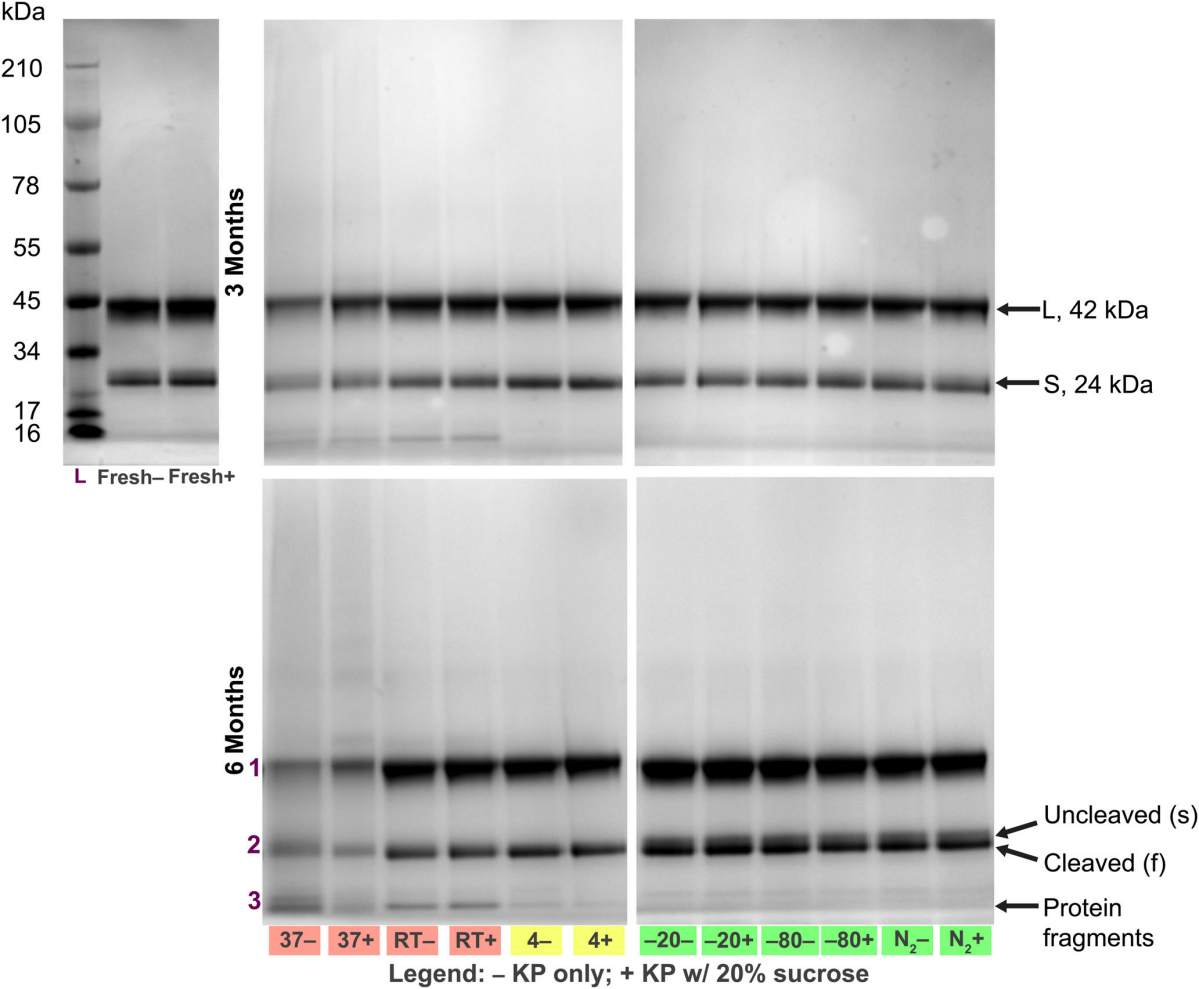
Sodium dodecyl sulfate‐polyacrylamide gel electrophoresis. Heat denatured cowpea mosaic virus (CPMV) particles stained with Coomassie Brilliant Blue and imaged under white light. Fresh CPMV (leftmost) is compared to particles stored for 3 months (top) and 6 months (bottom). Additional time points can be found in Figure S3. Bands marked “1,” “2,” and “3” for K: Fresh, 37°C, room temperature (RT), and 4°C were excised and analyzed by liquid chromatography with tandem mass spectrometry to evaluate peptide content (Figure S12). Red highlighting of 37°C and RT lanes indicates detectable protein fragments; yellow on 4°C lane indicates bands in which there may be cleavage of the *S* protein but otherwise minimal visible protein fragments; and green indicates acceptable band patterns.

Next, we analyzed the electrophoretic behavior of various CPMV samples using native and denaturing gels. In native gels, the two electrophoretic forms, denoted as slow (s) and fast (f) are detectable, and the RNA and protein co‐migrate (Figure 2). The s and f electrophoretic forms of CPMV are explained by loss of 24 amino acids from the C‐terminus of the *S* protein; the cleavage occurs in planta or during storage.^12^ The s and f forms of *S* (24 kDa) are also detectable by SDS‐PAGE; the *L* (42 kDa) protein does not exhibit such cleavage products (see Figure 3). Native and denaturing gels indicate that frozen samples are stable (Figures 2 and 3). In stark contrast, in samples stored at 37°C and RT, CP degradation, RNA loss, and RNA degradation, as well as aggregation, were apparent within 1 month of storage, with this effect becoming more profound over time. In these samples, native gels show free RNA and significant particle aggregation with slower mobility bands and RNA and protein detected in the gel pockets (Figures 2, S3, and S4). CPMV instability was also apparent by the increased presence of low‐molecular weight protein bands (Figures 3 and S5); LC–MS/MS analysis confirmed these protein fragments to be peptides from both CPMV *S* and *L* (Figure S6). Samples that were incubated at 4°C exhibited some signs of changes, albeit data were not necessarily consistent with degradation: particle aggregation or loss of RNA was not apparent; however, the preparation, which started with a mixture of CPMV‐s and CPMV‐f, turned over to only contain CPMV‐s, which is distinct from the cold storage samples that maintained the mix of CPMV‐s and CPMV‐f. This is consistent with colder conditions slowing energy‐dependent biological processes. We also extracted RNA from particle preparations and analyzed purified RNA using the bioanalyzer (Figure S7). Data were consistent and indicated significant RNA loss and degradation for samples stored at 37°C and RT. Samples stored at 4 and −80°C yielded less than 100 ng/μL RNA, but along with −20°C and N_2_ samples, still showed characteristic RNA bands similar to those found in fresh CPMV (the lower yields are likely explained by artifacts of sample processing rather than degradation).

**FIGURE 4:**
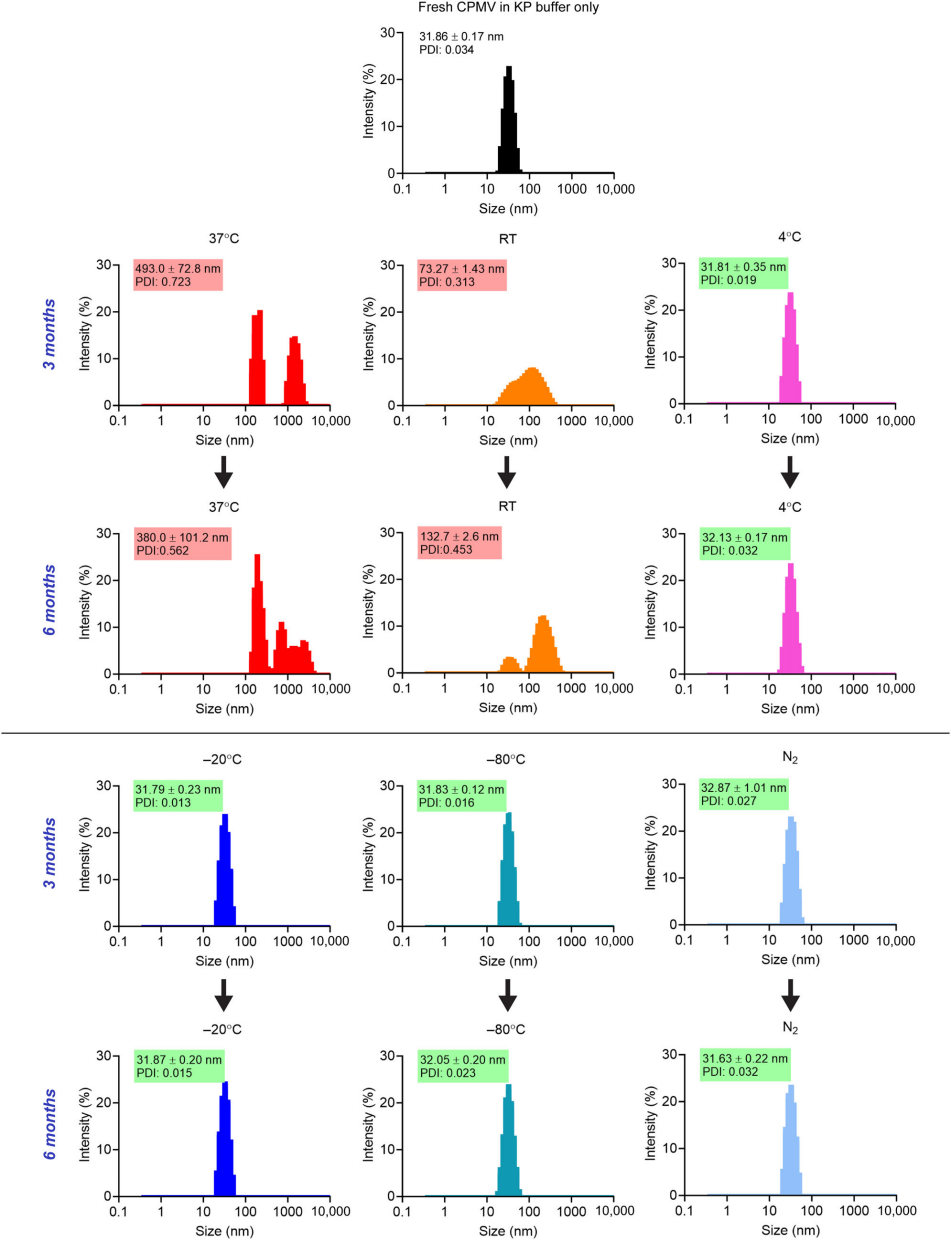
Dynamic light scattering (DLS). Size and degree of aggregation of cowpea mosaic virus (CPMV) were measured by DLS. Sizes are reported as the mean of three technical replicates ± standard deviation. Low polydispersity index (PDI) values indicate monodisperse particles and high values indicate a broad size distribution. Red highlights signify aggregation and high polydispersity, while green highlights indicate acceptable size and high monodispersity. Additional time points can be found in Figures S4 and S5. KP, potassium phosphate.

Next, we used a combination of DLS, fast protein liquid chromatography (FPLC), and TEM to analyze the sizing, aggregation state, and degradation products of the CPMV formulations. For CPMV, whose hydrodynamic radius is 30 nm, aggregation is best quantified via DLS (Figure 4). A typical DLS plot for CPMV shows a narrow size distribution centered around 30 nm with a low polydispersity index (PDI <0.2). This is consistent with highly monodisperse formulations, as expected for a biologic nanoparticle. In general, samples with a PDI <0.05 are considered highly monodisperse, while those with PDI values >0.7 are characterized by heterogeneity.^26^ CPMV stored at 4°C or colder generally maintained a narrow size distribution and low PDI (between 31 and 34 nm, PDI <0.1) through 9 months of testing (Figures S8 and S10). There was one N_2_ sample at 1 week of storage with an increased radius of 44.09 nm and PDI 0.104; however, since the radius returned to the expected range for the remainder of the study, we attribute this to a technical inconsistency rather than true aggregation. At 37°C, CPMV quickly aggregated, with a secondary peak appearing centered around 1000 nm after just 1 week of storage; the high degree of heterogeneity was also reflected by a high PDI of 1.000. The fraction of 30 nm particles decreased over time, with only large aggregates present by 6 months (380 nm, 0.562 PDI). A similar trend was observed for CPMV stored at RT—albeit the rate of aggregate formation was slower. Large CPMV aggregate appeared at the 3 months mark with a size of 73 nm (PDI = 0.313) and 132 nm (PDI = 0.453) at 3 and 6 months, respectively. Protein aggregation is the most common indicator of physical degradations, and based on standards for intravenous antibody products from the World Health Organization, the aggregate level should be less than 5%.^27^ While CPMV is neither an antibody nor is the intended administration route intravenous (it is a candidate for intratumoral immunotherapy), this guideline gives a good reference point for biologics.

Interestingly, the sucrose additive increased the hydrodynamic radius of CPMV immediately after preparation and across all storage temperatures, ranging from ~42 to 73 nm for 4°C and colder for all time points (Figures S9 and S10). The sucrose appeared to have some interaction with the CPMV, to the point of creating a corona to increase the hydrodynamic radius. However, in refrigerated and colder temperatures, the radius decreased over time, which was a somewhat unexpected finding. This may indicate that sucrose first coats the particle, but over time some structural change occurs within the sucrose layer; this will require further interrogation in a future study.

**FIGURE 5:**
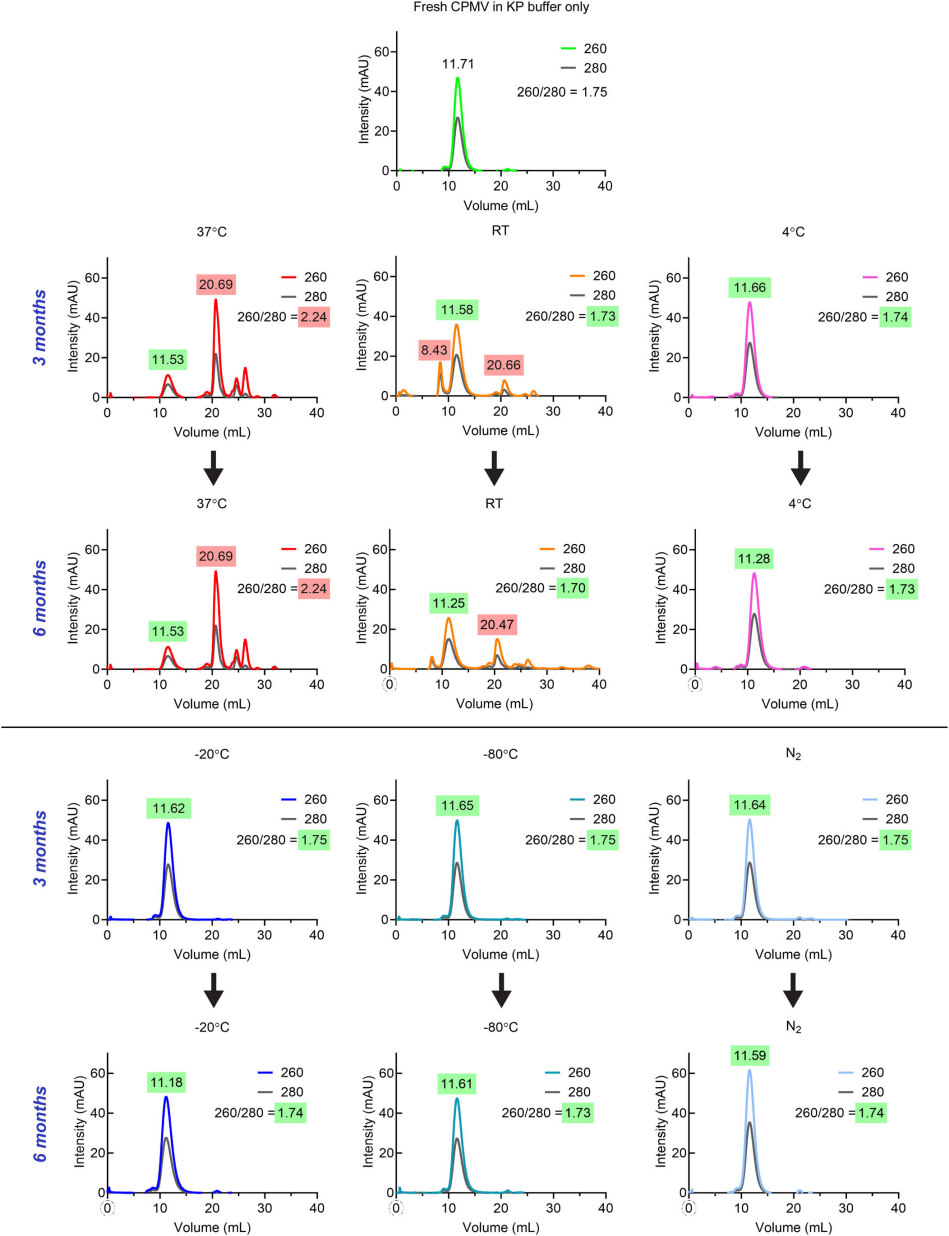
Fast protein liquid chromatography (FPLC). Cowpea mosaic virus (CPMV) particle size and integrity were further evaluated by FPLC using a Superose 6 Increase 10/300 GL column. The characteristic elution profile for CPMV is a single peak ~11.7 mL, highlighted in green, and any peaks appearing before or after this volume are indicative of aggregation (former) or broken particles (latter), highlighted in red. Absorbances at 260 nm (RNA) and 280 nm (protein) are expected to co‐elute at a ratio (*A*260/280) of 1.8 for intact particles, with values between 1.7 and 1.9 emphasized in green and values outside this range in red. Additional time points can be found in Figures S6 and S7. KP, potassium phosphate; RT, room temperature.

The trends observed with DLS were largely consistent in FPLC analysis; FPLC is not only a measure of aggregation but will also provide insights into degradation products. Intact CPMV elutes at 11–12 mL from the Superose 6 Increase column with the characteristic 260:280 ratio of 1.7–1.8. Frozen and 4°C samples matched the elution profiles of freshly prepared CPMV (Figure 5). In stark contrast, after just 1 week, CPMV stored at 37°C showed additional degradation peaks at approximately 20 mL—and the effects were more profound the longer the samples were exposed to higher temperatures (Figures S11 and 12). By 6 months at 37°C, there was a minimal amount of intact CPMV left, with most of the sample appearing as disassembled CPs and/or protein fragments eluting at ~20 mL. Furthermore, as degradation progressed, the 260:280 ratio of RNA:protein increased to over 2.0, far outside the acceptable range for purity and indicative of RNA loss. The RT samples followed a similar, yet delayed trend: by 3 months, additional degradation peaks at 20 mL appeared, as well as an aggregation peak around 8.4 mL.

**FIGURE 6:**
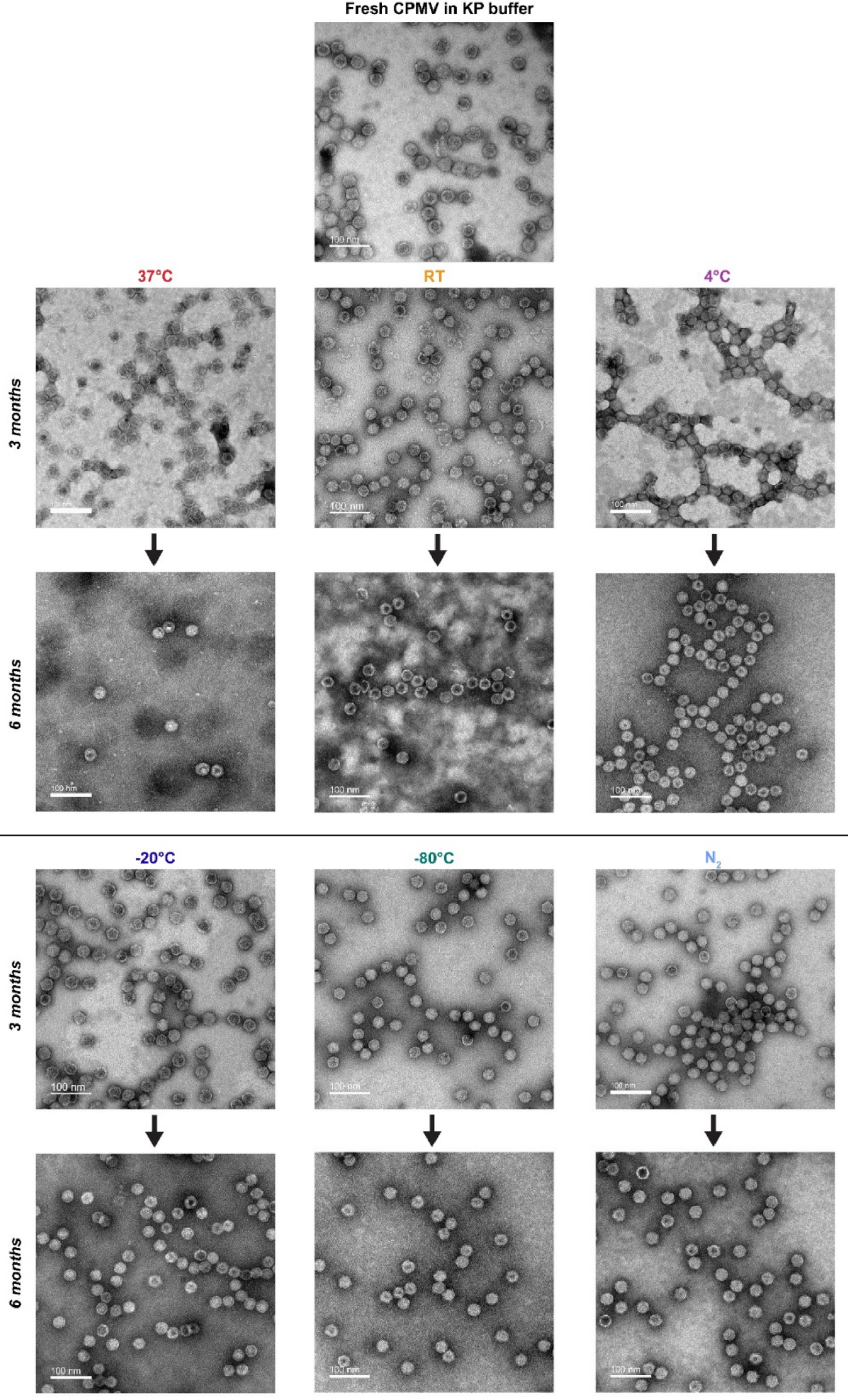
Transmission electron microscopy (TEM). Negatively stained cowpea mosaic virus (CPMV) particles were imaged via TEM at 80,000× magnification to confirm size and to inspect for visual cues of degradation. The scale bar is 100 nm. Additional time points can be found in Figures S8 and S9. KP, potassium phosphate; RT, room temperature.

Electron microscopy profiling allowed us to visually confirm the level of intactness implied by the other characterization techniques (Figures 6, S13, and S14). For −20°C, −80°C, and N_2_ stored CPMV, particles were perceptibly fully intact throughout the duration of the study. Four degrees Celsius appeared stable until 9 months; however, some broken CPMV could be identified. With RT storage, broken CPMV could be seen by 1 month, and at 37°C as early as 1 week. By 6 months, it became difficult to find intact particles from 37°C to image. The findings here follow the trends detailed above by all other characterization methods.

The combination of characterization methods consistently showed frozen CPMV to be physically stable; RT and 37°C not stable, and 4°C somewhat variable. Although sucrose is commonly used in the biopharmaceutical industry to increase the chemical and thermal stability of proteins, plant viruses such as CPMV exceed the weight and size dimensions of proteins typically used in pharmaceutical applications today.^22^, ^28^ It was yet to be seen if sucrose could also protect CPMV against degradation, but our data show minimal structural differences between CPMV stored with sucrose versus without, indicating sucrose is not an ideal preservative in this case. Based on the physical characterization, the recommendation would be to store CPMV at −20°C; this is unlike past recommendations and literature that indicate storage at 4°C^12^ or denaturation at −20°C.^29^

### 3.2 Effects of freeze–thaw on stability

When biologics or any sample is stored at 20°C (or colder), it is important to ensure stability after free–thaw cycles. Therefore, we tested the physical stability of CPMV after 3 months of storage in −20°C followed by 10 cycles of thawing for 24 h at RT and then refreezing for 24 h. Characterization data (Figure S15) establishes that agarose and SDS‐PAGE gel patterns match those of −20°C CPMV that was *not* subjected to freeze–thaw; the 260:280 ratio, DLS peak, and FPLC peak all maintain correlation to the standard of freshly stored CPMV; also, TEM was consistent with the presence of structurally sound CPMV with minimal broken particles detectable. These data illustrate that for up to 10 freeze–thaw cycles, the structural stability of CPMV is not impacted.

### 3.3 Biological activity of CPMV after long‐term storage

**FIGURE 7:**
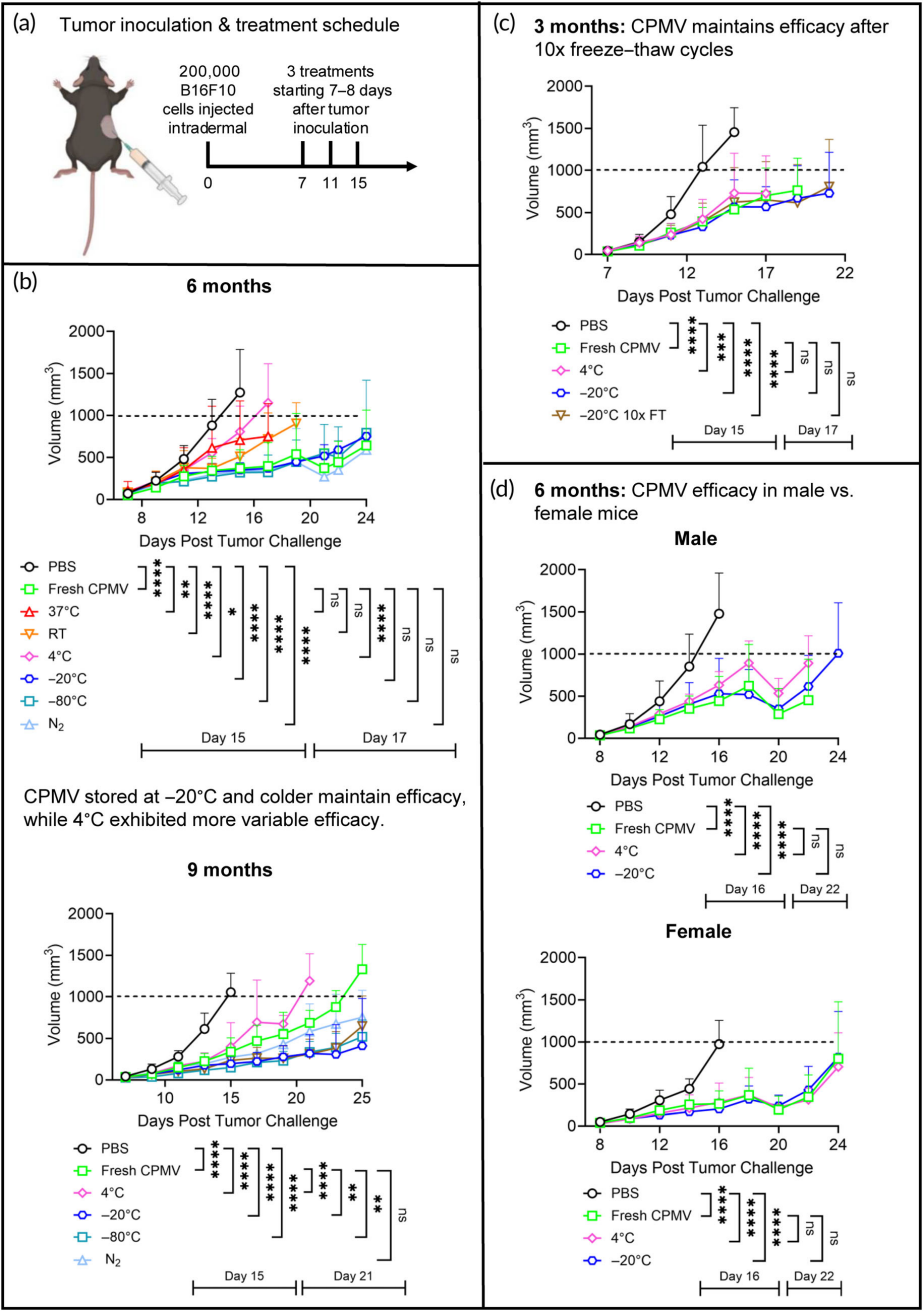
Intratumoral treatment against B16F10 murine melanoma using fresh cowpea mosaic virus (CPMV) versus aged CPMV stored in multiple conditions. (a) Male C57BL/6 mice were inoculated with 200,000 B16F10 cells intradermally on Day 0. Mice were then treated intratumorally starting on Days 7 and 8 with 100 μg of either fresh CPMV or aged CPMV stored at 37°C, RT, 4°C, −20°C, −80°C, or liquid N_2_. Mice received a total of three treatments every 4 days after the first treatment. Mouse image made in BioRender.com (b) Tumor growth after storage for 6 months (top) and 9 months (bottom) was monitored by tumor volume (pictured) and body weight (Figure S15). Mice were euthanized when tumor volume reached 1000 mm^3^. Data are means ± standard deviation (*n* = 5). Growth curves end when there are less than three mice remaining alive in a given group to avoid significant changes in the growth curve average. The full outcome is completely tracked by survival curves (Figure S15). (c) Tumor growth of a separate batch of CPMV aged for 3 months, including CPMV that had been subjected to 10 freeze–thaw cycles (see also Figure S15). (d) Tumor growth in male versus female mice after 6 months of storage. Individual growth curves and survival curves are shown in Figure S16. Statistical analysis of growth curves was calculated using a two‐way analysis of variance (ANOV and the significance of survival curves was calculated using the Mantel–Cox log rank test, with ****p < 0.0001, ***p < 0.0002, **p < 0.0021, *p < 0.0332, and ns = *p* < 0.1234.

Following 6 and 9 months of storage, we evaluated the biological activity of the CPMV samples; first by testing efficacy in a dermal melanoma mouse model and second by evaluating cytokine stimulation in human PBMCs. Male and female C57BL6 mice were inoculated with 2.0 × 10^5^ B16F10 cells intradermally on Day 0. Once tumors reached at least 20 mm^3^ (which typically is around Day 7), mice were treated intratumorally with fresh CPMV or CPMV stored at 37°C, RT, 4°C, −20°C, −80°C, or N_2_ for 3, 6, or 9 months (100 μg CPMV/20 μL PBS, with 20 μL PBS serving as NC). First, we compared efficacy only in male mice (Figure 7a).

At 6 months, by 20 days post‐tumor challenge, there was an evident split in efficacy between the PBS control group and mice treated with CPMV stored at 37°C, RT, or 4°C, versus fresh CPMV and CPMV stored at −20°C, −80°C, or N_2_ (Figure 7b), indicating that all frozen samples matched the efficacy of freshly prepared CPMV. Fresh and frozen‐stored CPMV similarly slowed tumor growth in comparison to the control group, and by the end of the study, there was no significant difference in survival between mice treated with fresh CPMV and −20°C, −80°C, or N_2_‐stored CPMV (survival curves and individual tumor growth curves can be found in Figure S16). Conversely, there was a difference in survival comparing fresh CPMV versus RT‐stored CPMV (*p < 0.0332) and between fresh CPMV and samples stored at 37°C or 4°C (**p < 0.0021). It was not unexpected that CPMV stored at 37°C or RT would have decreased antitumor efficacy, as there was significant particle degradation and aggregation. However, it was somewhat surprising that CPMV stored at 4°C also displayed diminished potency—considering only subtle differences in structure were noted by denaturing gels and TEM.

The efficacy study was repeated at the 9‐month time point (Figures 7b and S16). To keep the animal numbers to a minimum, we omitted CPMV samples stored at 37°C and RT because these samples show significant signs of degradation and little to no efficacy at the 6‐month time point. Consistent with the 6‐month timepoint, the frozen samples showed comparable efficacy to fresh CPMV, thus attesting to the stability and biological activity of CPMV stored frozen (−20°C up to liquid N_2_). The sample stored at 4°C showed efficacy albeit with diminished potency.

To probe the efficacy of the 4°C‐storage sample further, we prepared a new batch of CPMV and stored one portion of the batch at 4°C and the other at −20°C. At the 3‐month time point, we then compared efficacy of fresh versus 4 and −20°C‐stored samples; we also included a sample that was stored at −20°C and then underwent 10 freeze–thaw cycles (Figures 7c and S16). Physical characterization showed intact CPMV for all tested storage conditions (not shown here); the in vivo efficacy study also showed matched efficacy of the samples in comparison. Thus, data indicate that samples stored frozen maintain efficacy and that samples stored at 4°C may exhibit a greater degree of variability. We hypothesize that the amino acid loss on the *S* protein, as seen in the SDS‐PAGE and agarose gels for the first batch of 4°C‐stored samples, may impact the efficacy of CPMV. Samples stored at 4°C in this repeat study did not show the same extent of *S* protein cleavage compared to the first batch. Hence, this difference may explain why in the second study, 4°C‐stored samples matched the efficacy of fresh CPMV, but samples with a greater extent of *S* protein cleavage used in the first study, showed diminished efficacy. This further indicates that CPMV stored in refrigeration can maintain stability and efficacy but may also result in a higher degree of variability due to subtle structural changes.

Finally, we included sex as a variable and analyzed the efficacy of CPMV in male versus female mice bearing B16F10 dermal tumors (Figures 7d and S17). Though tumor growth was slightly delayed in female mice versus male mice, there was no significant difference in survival between the two fresh CPMV groups (mean survival of 22 days for male, 26 days for female mice). There was additionally no significant variation in survival of 4 or −20°C‐stored CPMV when compared to fresh CPMV or between the two mouse genders. While this study indicates there are differences in tumor growth rates, the data indicate the potency of CPMV is not affected by sex, though this may need to be verified by additional studies.

**FIGURE 8:**
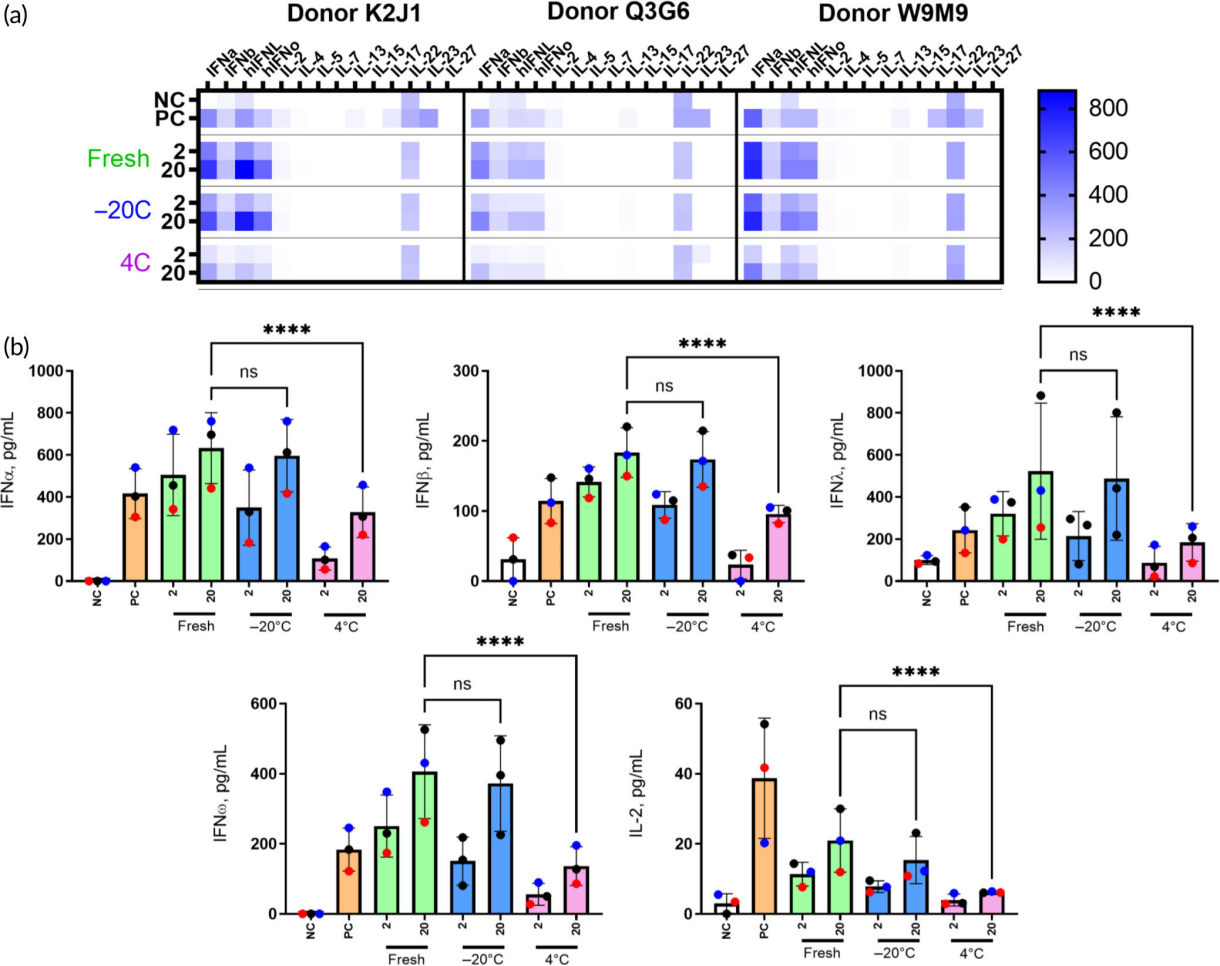
Cytokine induction by fresh versus aged cowpea mosaic virus (CPMV). Peripheral blood mononuclear cells from healthy human donor volunteers were exposed to controls or CPMV for 24 h, and the activation of cytokine responses was assessed using a 14‐plex panel. (a) Heatmap summary of the data demonstrating overall cytokine responses to fresh CPMV or CPMV stored for 1 year at a nominal temperature of 4 or −20°C. Shown is the mean response (*N* = 3) of each sample; the intensity of the blue color represents the magnitude of the cytokine response; the scalebar shows color intensity corresponding to cytokine concentrations in pg/mL. (b) Individual graphs of the five cytokines from (a) consistently induced in all donors. Each bar shows the mean response and standard deviation of data obtained from three healthy donors. Each dot shows the mean responses (*N* = 3) of cells from individual donors as follows: black—Donor K2J1; red—Donor Q3 G6, and blue—Donor W9M9. NC = negative control (PBS); PC = positive control (5 μg/mL ODN2216 and 10 μg/mL PHA‐M). Statistical significance between fresh, −20, and 4°C samples was calculated using two‐way ANOVA with ****p < 0.0001 and ns = *p* < 0.1234. IFN, interferon.

Finally, to correlate efficacy with mechanism of action, we analyzed cytokine responses to CPMV in human PBMC cultures using 14‐plex ELISA. Specifically guided by the physical characterization studies and efficacy studies in mice, we selected fresh CPMV as well as CPMV stored at 4 and −20°C for 1 year (Figure 8). Type I (IFNα, IFNβ, and IFNω) and type III (IFN λ) IFNs and IL‐2 were detected in all samples (Figure 8, individual cytokine plots can be found in Figure S18). These data suggest the activation of plasmacytoid dendritic cells (pDCs) and T‐cells by CPMV and is consistent with earlier reports describing similar immunostimulatory responses to RNA‐containing animal viruses (reviewed in Refs. 30, 31, 32), RNA‐based nucleic acid nanoparticles, and therapeutic RNA^33^, ^34^ and CpG oligonucleotides.^35^, ^36^ Cytokine levels induced by CPMV were comparable to and, in some cases, stronger than that induced by the assay PC (Figure 8b; compare IFN response observed with fresh and stored at −20°C CPMV to that in PC). These data suggest that CPMV at tested concentrations possesses IFN‐inducing activity superior to that of ODN2216, a CpG oligonucleotide identified to selectively and potently activate pDCs.^35^ The magnitude of the response was lower in the refrigerated (4°C) sample versus fresh CPMV, and the sample stored at −20°C. These data are consistent between cultures derived from different donors and suggest that −20°C provides more optimal storage conditions for CPMV in situations where using freshly isolated viral particles is not feasible.

Altogether, physical characterization data showed all frozen CPMV samples remained stable; there were subtle differences noted for the refrigerated (4°C) sample, which was also reflected by more variable efficacy in tumor mouse models and reduced immunomodulatory properties in cyto/chemokine analysis. While the inherent instability of the 4°C sample was not apparent, the capsid changed from a mixed s/f electrophoretic form to almost exclusively f‐form, as well as a minor proportion of not fully intact particles visible in TEM images at 9 months. Although previous studies note that the 24‐amino acid cleavage does not affect particle stability or infectivity, it may be this subtle structural difference that can be attributed to the variability in the biological assays.^37^

## 4 CONCLUSIONS

Our data identifies the ideal storage temperature of CPMV to be −20°C or colder. While this work identifies a simple KP buffer to be sufficient, future studies may also want to perform buffer and preservative screening to identify a more ideal storage formulation. Previous work intentionally altered CPMV through RNA removal (eCPMV) or inactivation (UV crosslinking and chemical inactivation), identifying native, RNA‐laden CPMV as the most efficacious.^8^, ^38^, ^39^ The current study corroborates previous conclusions that the integrity of both the viral capsid and encapsidated RNA is essential to intratumoral immunotherapeutic efficacy, as evidenced by the lack of anti‐tumor potency by CPMV stored at 37°C and RT conditions, which degraded and lost RNA over time. However, eCPMV has been proven to maintain potency in canine studies in combination treatment regimens, indicating RNA loss does not entirely negate CPMV's positive impacts.^9^, ^10^, ^11^ Additionally, there may still be questions regarding the extent of capsid integrity necessary for full functionality; the potency of CPMV stored at 4°C was inconsistent, though characterization showed minimal differences from fresh or frozen‐stored CPMV. Future studies may want to explore these discrepancies, in particular the potential effect of the amino acid cleavage from the *S* CP that was observed here for 4°C‐stored CPMV. Importantly, we also found that multiple freeze–thaw cycles do not negatively affect the structural or functional stability of CPMV, which will allow for fewer concerns regarding long‐distance travel through the imperfect cold chain. Overall, data suggest that frozen conditions (−20°C or colder) lead to the best and most consistent results and maintain CPMV particle integrity and efficacy following extended periods of storage.

## CONFLICT OF INTEREST STATEMENT

The authors declare the following competing financial interest(s): Dr. Nicole F. Steinmetz is a co‐founder of, has equity in, and has a financial interest with Mosaic ImmunoEngineering Inc. Dr. Nicole F. Steinmetz is a co‐founder of, and serves as manager of Pokometz Scientific LLC, under which she is a paid consultant to Mosaic ImmunoEngineering Inc., Flagship Labs 95 Inc., and Arana Biosciences Inc. The other authors declare no potential conflict of interest.

## Supporting information


**Data S1.** Supporting information for additional timepoints of all characterizations can be found in Figures S1–S15. Survival curves and individual mouse tumor volume plots for all treatment studies are in Figures S16 and S17. Individual cytokine data are in Figure S18.

## Data Availability

Data supporting this study are available from the authors upon reasonable request.
