# Supplementary material for: Cowpea mosaic virus intratumoral immunotherapy maintains stability and efficacy after long‐term storage
Source: Bioeng Transl Med. 2024 Jul 7;9(6):e10693. doi: 10.1002/btm2.10693 (PMC11558193; doi:10.1002/btm2.10693)
Supplement: Supplementary file 1 — Data S1. Supporting information for additional timepoints of all characterizations can be found in Figures S1–S15. Survival curves and individual mouse tumor volume plots for all treatment studies are in Figures S16 and S17. Individual cytokine data are in Figure S18. [file BTM2-9-e10693-s001.docx]

**Supporting Information**

**Cowpea Mosaic Virus Intratumoral Immunotherapy Maintains Stability and Efficacy after Long-Term Storage**

Authors:

Andrea Simms^1,4,5,8^, Zhongchao Zhao^1,4,5,8^, Edward Cedrone^9^, Marina Dobrovolskaia^9^, Nicole F. Steinmetz^1,2,3,4,5,6,7,8^

^1^Department of NanoEngineering, University of California, San Diego, 9500 Gilman Dr., La Jolla, CA, 92093 USA

^2^Department of Bioengineering, University of California, San Diego, 9500 Gilman Dr., La Jolla, CA, 92093 USA

^3^Department of Radiology, University of California, San Diego, 9500 Gilman Dr., La Jolla, CA, 92093 USA

^4^Center for Nano-ImmunoEngineering, University of California, San Diego, 9500 Gilman Dr., La Jolla, CA, 92093 USA

^5^Moores Cancer Center, University of California, San Diego, 9500 Gilman Dr., La Jolla, CA, 92093 USA

^6^Institute for Materials Discovery and Design, University of California, 9500 Gilman Dr., La Jolla, CA, 92093 USA

^7^Center for Engineering in Cancer, Institute for Engineering in Medicine, University of California, 9500 Gilman Dr., La Jolla, CA, 92093 USA

^8^Shu and K.C. Chien and Peter Farrell Collaboratory, University of California, 9500 Gilman Dr., La Jolla, CA, 92093 USA

^9^Nanotechnology Characterization Lab., Cancer Research Technology Program, Frederick National Laboratory for Cancer Research sponsored by the National Cancer Institute, 8560 Progress Drive, Frederick, MD 21701 USA

^#^Corresponding author: [nsteinmetz@ucsd.edu](mailto:nsteinmetz@ucsd.edu)

**Production of CPMV**

All buffers were stored in glassware that had been baked at 200°C for at least 4 hours and autoclaved, except those containing sucrose, to minimize endotoxin contamination. Frozen, CPMV-infected cowpea plant leaves stored at -70°C were homogenized using a standard blender with 3 volumes of chilled 0.1 M potassium phosphate (KP) buffer pH 7.0, then filtered through 2 layers of Miracloth (MilliporeSigma, St. Louis, MO, USA). The crude plant homogenate was then centrifuged using a Beckman Coulter Avanti J-E centrifuge and JLA 10.500 rotor at 18,500 g for 20 min at 4°C. The supernatant was then mixed with 0.7 volumes of 1:1 (v/v) chloroform:1-butanol (both ThermoFisher Scientific, Waltham, MA, USA) on ice for 30 min. This mixture was then centrifuged using a Beckman Coulter Avanti J-E centrifuge (Brea, CA, USA) and JLA 10.500 rotor at 6,600 g for 10 min at 4°C, and the dark yellow upper aqueous layer was collected. 0.2 M NaCl (ThermoFisher Scientific, Waltham, MA, USA) and 10% (w/v) PEG (MW 8,000, ThermoFisher Scientific, Waltham, MA, USA) were added, the mixture was stirred on ice for 30 min, then mixed overnight in 4°C. The mixture was then centrifuged using a Beckman Coulter Avanti J-E centrifuge and JLA 16.250 rotor at 30,000 g for 15 min at 4°C. The pellet was resuspended in 5 mL chilled 0.01 M KP buffer pH 7.0, and the resuspension was centrifuged at 13,500 g for 15 min at 4°C using a Beckman Coulter Avanti J-E centrifuge (Brea, CA, USA) and JLA 16.250 rotor. The supernatant was collected, and the pellet was resuspended again and centrifuged again for a second recovery. A sucrose gradient was prepared in Beckman Coulter Ultraclear ultracentrifuge tubes (Brea, CA, USA) by layering 12 mL 40% sucrose (w/v) underneath 12 mL 10% (w/v) sucrose (both in 0.05 M KP buffer pH 7.0), then running on a Gradient Master 108 (BioComp Instruments, Inc., Fredericton, NB, CA) using the 10-40% (w/v) gradient program for SW32 rotor provided by BioComp. The supernatant of the previous centrifugation was then placed on top of the sucrose gradient, and this solution was then ultracentrifuged using a SW32 Ti rotor at 28,000 rpm for 2 hours at 4°C. With the Ultraclear tubes placed over a flashlight, the middle two light scattering bands were collected and placed in new capped ultracentrifuge tubes (Beckman Coulter, Brea, CA, USA). These bands were ultracentrifuged using a Beckman Optima L-90K ultracentrifuge (Brea, CA, USA) and a 50.2 Ti rotor at 42,000 rpm for 2 hours at 4°C, and the supernatant discarded. The pellets were resuspended in 1 mL 0.1 M KP buffer pH 7.0 and placed on a shaker rack in 4°C overnight.

**Characterization Methods**

***Ultraviolet – Visible Spectroscopy (UV-Vis).*** NanoDrop UV-Vis (NanoDrop 2000, ThermoFisher Scientific, Waltham, MA, USA) was used determine the concentration of CPMV samples (εCPMV at 260 nm = 8.1 mL mg^-1^ cm^-1^) and to obtain the 260/280 ratio as a measure of purity and RNA quality: intact CPMV particles have a 260:280 ratio of 1.7-1.8, while RNA-free preparations of CPMV have a 260:280 ratio of 0.6-0.7.

***Size Exclusion Chromatography (SEC).*** CPMV samples diluted to 1 mg/mL were run on an Äkta Explorer FPLC (Cytiva, Marlborough, MA, USA) through a Superose 6 Increase 10/300 GL column, with 0.1 M KP buffer pH 7.0 as the mobile phase. An injection volume of 100 µL and flow rate of 0.5 mL/min were used, and absorbance at 260 and 280 nm were recorded.

***Dynamic Light Scattering (DLS).*** A Zetasizer Nano ZSP/Zen5600 (Malvern Panalytical, Malvern, UK) instrument was used for all DLS measurements. 3 technical replicates of 200 µL CPMV at 1 mg/mL were measured 25°C.

***Sodium Dodecyl Sulfate – Polyacrylamide Gel Electrophoresis (SDS-PAGE).*** Samples were prepared for SDS-PAGE by mixing 20 µL 1 mg/mL CPMV with 4 µL 10x reducing agent (Invitrogen, Waltham, MA, USA), 10 µL 4x lithium dodecyl sulfate buffer (Life Technologies, Carlsbad, CA, USA), and 6 µL 0.1 M KP buffer pH 7.0, then heated at 95°C for 10 min. 10 µL SeeBlue™ Plus2 Pre-stained Protein Standard (Invitrogen, Waltham, MA, USA) and 20 µL each sample were loaded onto a NUPAGE 4-12% Bis-Tris protein gel (ThermoFisher Scientific, Waltham, MA, USA). Each run was performed using 1x NuPAGE MOPS running buffer (Invitrogen, Waltham, MA, USA) at 200 V, 120 mA, and 25 W for 35 min. Gels were stained with Coomassie Brilliant Blue (stock solution: 0.25% [w/v] Coomassie Brilliant Blue, 10% [v/v] acetic acid, 45% [v/v] water, and 45% [v/v] methanol, stock diluted 10x in water, all ThermoFisher Scientific, Waltham, MA, USA) and then imaged in visible light on a ProteinSimple AlphaImager system (San Jose, CA, USA).

***Native agarose gel electrophoresis.*** Samples for agarose gel were prepared by mixing 12 µL 1 mg/mL CPMV with 4 µL 6x Gel Loading Dye Purple (New England Biolabs, Ipswich, MA, USA) and 8 µL 0.1 M KP buffer pH 7.0. 15 µL of each sample was loaded into a 1.2% (w/v) agarose gel stained with GelRed Nucleic Acid Gel Stain (Biotium, Fremont, CA, USA), then run at 80 V and 300 mA for 40 minutes, with chilled 0.01 M KP buffer pH 7.0 used as the running buffer. Gels were first imaged under UV light to visualize the RNA, then stained with Coomassie Brilliant Blue (same as SDS-PAGE stain above) and imaged under visible light to detect protein band(s). A ProteinSimple AlphaImager system (San Jose, CA, USA) was used for all gel imaging.

***RNA Extraction and Bioanalyzer.*** RNA was extracted from 50 µg CPMV using a Quick-RNA Miniprep Plus Kit (Zymo Research, Irvine, CA, USA). Quantification of RNA was accomplished with UV-Vis spectroscopy and concentrated samples were diluted to 100 ng/µL (though some samples produced much less than 100 ng/µL). 260/280 and 260/230 ratios were calculated. RNA samples were further examined with the RNA 6000 Nano Assay kit (Agilent, Santa Clara, CA, USA) on an Agilent 2100 Bioanalyzer, using the manufacturer’s protocol.

***Transmission Electron Microscopy (TEM).*** TEM samples were prepared by applying 4 µL sample (0.5 mg/mL CPMV in 0.1 M KP buffer pH 7.0) onto a glow-discharged 400-mesh Cu grid (10 nm carbon support film, Electron Microscopy Sciences, Hatfield, PA, USA) for 30 s. The sample was then blotted with filter paper, rinsed with 4 µL Milli-Q water, and immediately blotted again. 4 µL of 0.75% (w/v) uranyl formate (Electron Microscopy Sciences, Hatfield, PA, USA) was applied for 30 s, then again blotted with filter paper. The grids were then allowed to air dry for at least 24 hours. A Jeol 1400Plus Transmission Electron Microscope (Peabody, MA, USA) was used to collect images at 50,000x and 80,000x magnifications.

***Liquid Chromatography with tandem mass spectrometry (LC-MS/MS).*** The L and S bands as well as additional lower molecular weight bands were excised from the gels using a razor blade submitted to the Biomolecular and Proteomics Mass Spectrometry Facility at UCSD for analysis. *In Gel Digest:* The gel slices were cut to 1 mm by 1 mm cubes and destained 3 times by first washing with 100 µL of 100 mM ammonium bicarbonate for 15 minutes, followed by addition of the same volume of acetonitrile (ACN) for 15 minutes. The supernatant was removed, and samples were dried in a Speedvac (ThermoFisher Scientific, Waltham, MA, USA). Samples were then reduced by mixing with 200 µL of 100 mM ammonium bicarbonate-10 mM DTT and incubated at 56°C for 30 minutes. The liquid was removed and 200 µL of 100 mM ammonium bicarbonate-55 mM iodoacetamide was added to gel pieces and incubated at RT in the dark for 20 minutes. After the removal of the supernatant and one wash with 100 mM ammonium bicarbonate for 15 minutes, the same volume of ACN was added to dehydrate the gel pieces. The solution was then removed, and samples were dried in a Speedvac. For digestion, enough solution of ice-cold trypsin (0.01 µg/µL) in 50 mM ammonium bicarbonate was added to cover the gel pieces and set on ice for 30 min. After complete rehydration, the excess trypsin solution was removed, replaced with fresh 50 mM ammonium bicarbonate, and left overnight at 37°C. The peptides were extracted twice by the addition of 50 µL of 0.2% formic acid and 5% ACN and vortex mixing at RT for 30 min. The supernatant was removed and saved. A total of 50 µL of 50% ACN-0.2% formic acid was added to the sample, which was vortexed again at room temperature for 30 min. The supernatant was removed and combined with the supernatant from the first extraction. The combined extractions were analyzed directly by liquid chromatography (LC) in combination with tandem mass spectroscopy (MS/MS) using electrospray ionization. *LC-MS/MS:* Trypsin-digested peptides were analyzed by ultra-high pressure liquid chromatography (UPLC) coupled with tandem mass spectroscopy (LC-MS/MS) using nano-spray ionization. The nanospray ionization experiments were performed using a Orbitrap fusion Lumos hybrid mass spectrometer (ThermoFisher Scientific, Waltham, MA, USA) interfaced with nanoscale reversed-phase UPLC (Thermo Dionex UltiMate™ 3000 RSLC nano System) using a 25 cm, 75-micron ID glass capillary packed with 1.7-µm C18 (130) BEH^TM^ beads (Waters Corporation, Milford, MA, USA).  Peptides were eluted from the C18 column into the mass spectrometer using a linear gradient (5–80%) of ACN at a flow rate of 375 μL/min for 1h. The buffers used to create the ACN gradient were: Buffer A (98% H_2_O, 2% ACN, 0.1% formic acid) and Buffer B (100% ACN, 0.1% formic acid). Mass spectrometry analysis was carried out using the Bruker TimsTOF Pro 2 (Bruker Nano, Inc., Tucson, AZ, USA). For the TimsTOF Pro 2 settings, the following parameters were adapted, starting from the PASEF method for standard proteomics. The values for mobility-dependent collision energy ramping were set to 95 eV at an inversed reduced mobility (1/*k_0_*) of 1.6 V s/cm^2^ and 23 eV at 0.73 V s/cm^2^. Collision energies were linearly interpolated between these two 1/*k_0_* values and kept constant above or below. No merging of TIMS scans was performed. Target intensity per individual PASEF precursor was set to 20,000. The scan range was set between 0.6 and 1.6 V s/cm^2^ with a ramp time of 166 ms. 14 PASEF MS/MS scans were triggered per cycle (2.57 s) with a maximum of seven precursors per mobilogram. Precursor ions in an *m/z* range between 100 and 1700 with charge states ≥3+ and ≤8+ were selected for fragmentation. Active exclusion was enabled for 0.4 min (mass width 0.015 Th, 1/*k_0_* width 0.015 V s/cm^2^). Protein identification and label free quantification was carried out using Peaks Studio 8.5 (Bioinformatics Solutions, Inc., Waterloo, ON, CA).

**
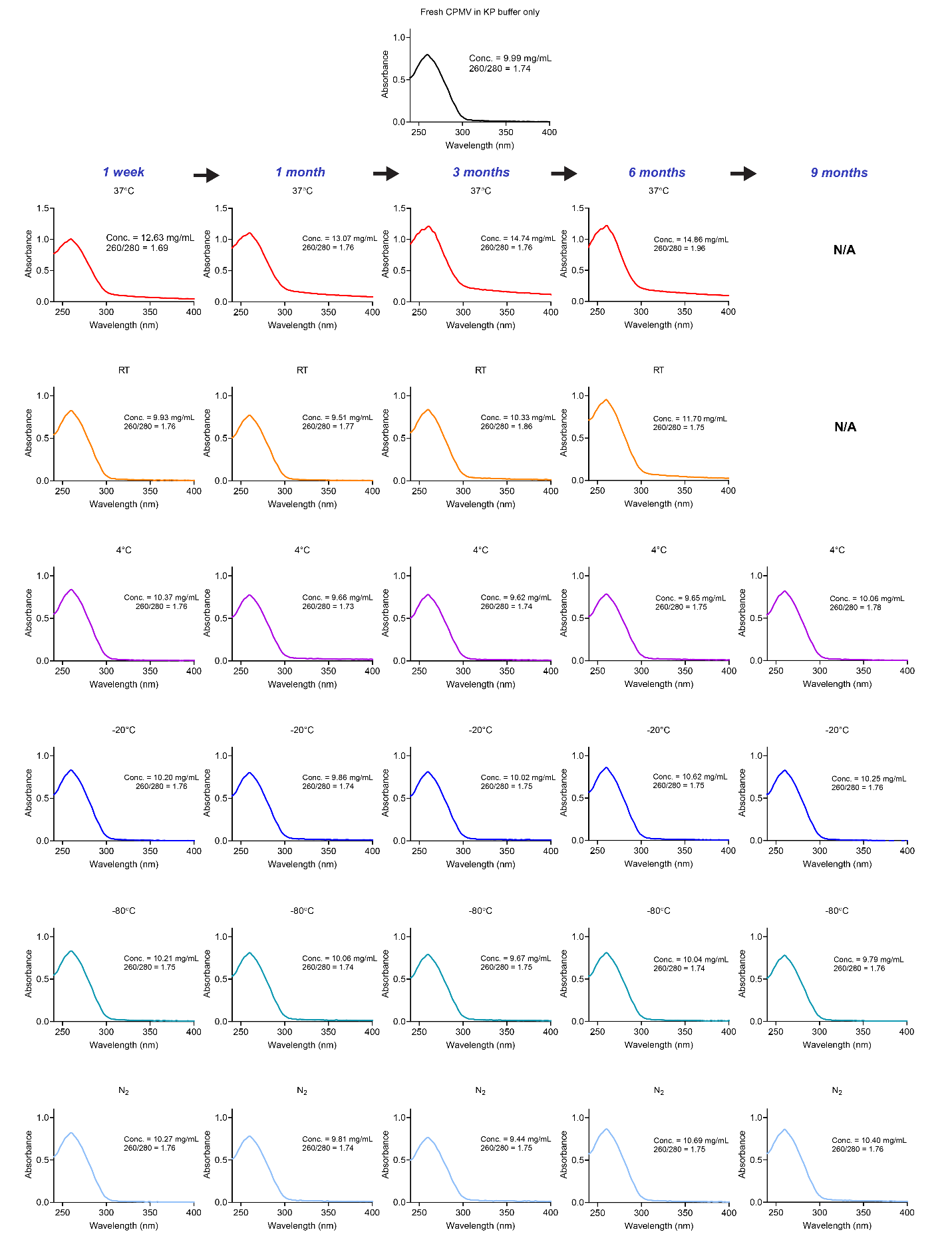
**

**Figure S1. UV-Vis spectroscopy, KP buffer.** Absorbance data from a NanoDrop 2000 spectrophotometer was used to calculate the RNA/protein (A260:280) ratio to measure purity and stability of CPMV. Pure and intact CPMV should have an A260:280 = 1.8.


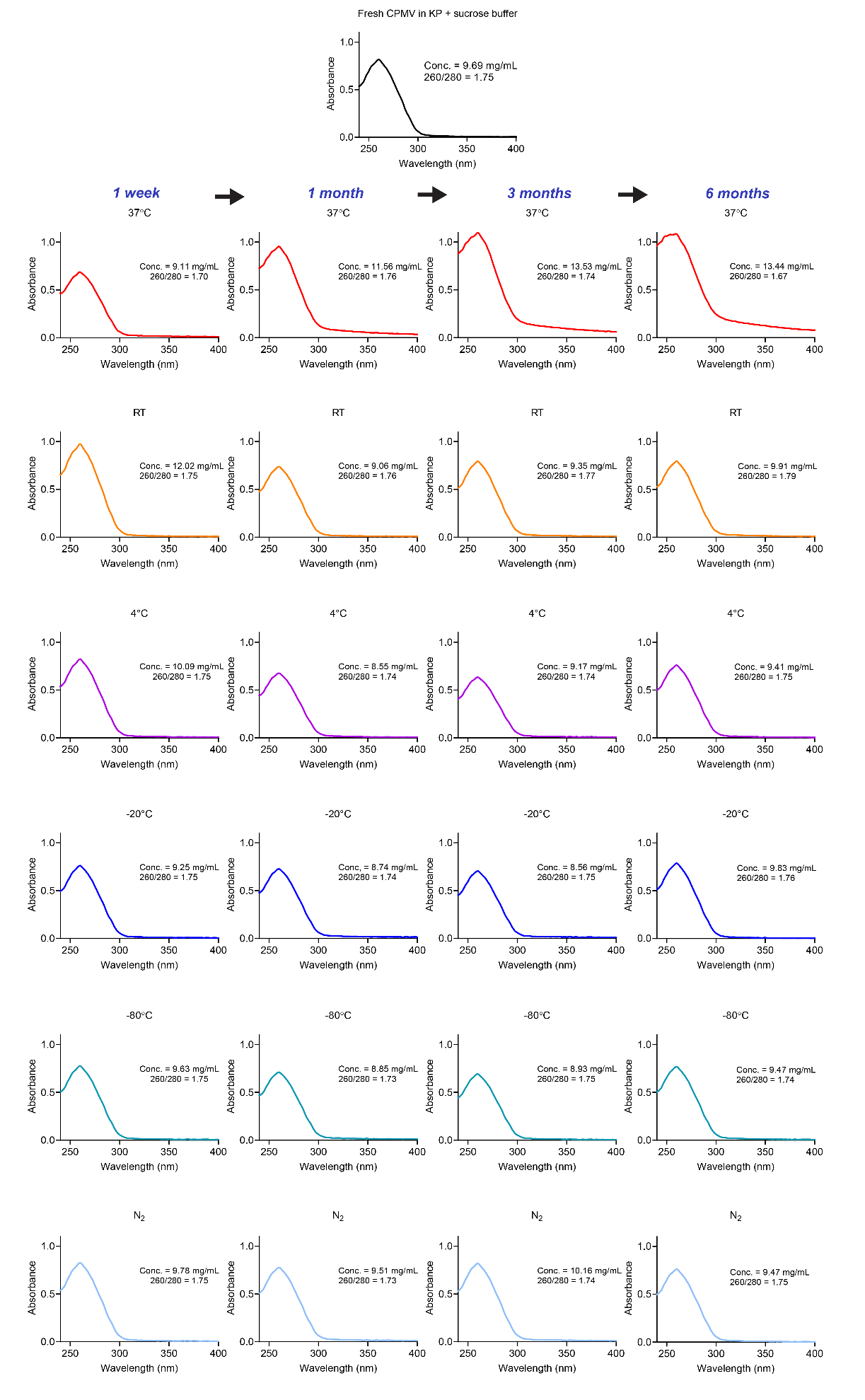


**Figure S2.** **UV-Vis spectroscopy, KP buffer + 20% sucrose.** Absorbance data from a NanoDrop 2000 spectrophotometer was used to calculate the RNA/protein (A260:280) ratio to measure purity and stability of CPMV. Pure and intact CPMV should have an A260:280 = 1.8.


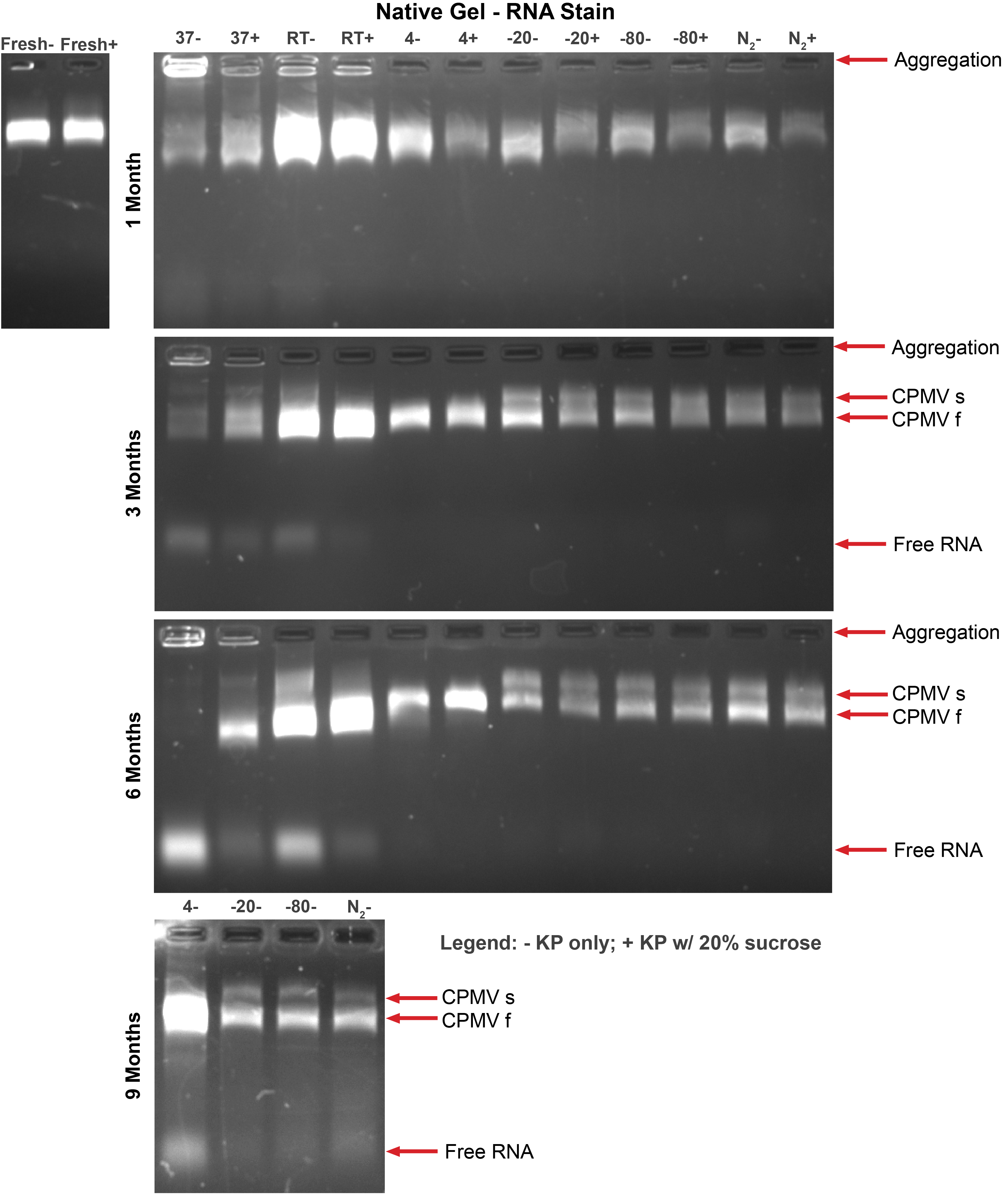


**Figure S3. Native agarose gel electrophoresis.** CPMV particles were stained with GelRed nucleic acid stain and imaged under UV light. A gel of fresh CPMV (leftmost) is compared to particles stored for 1 month, 3 months, 6 months, and 9 months.


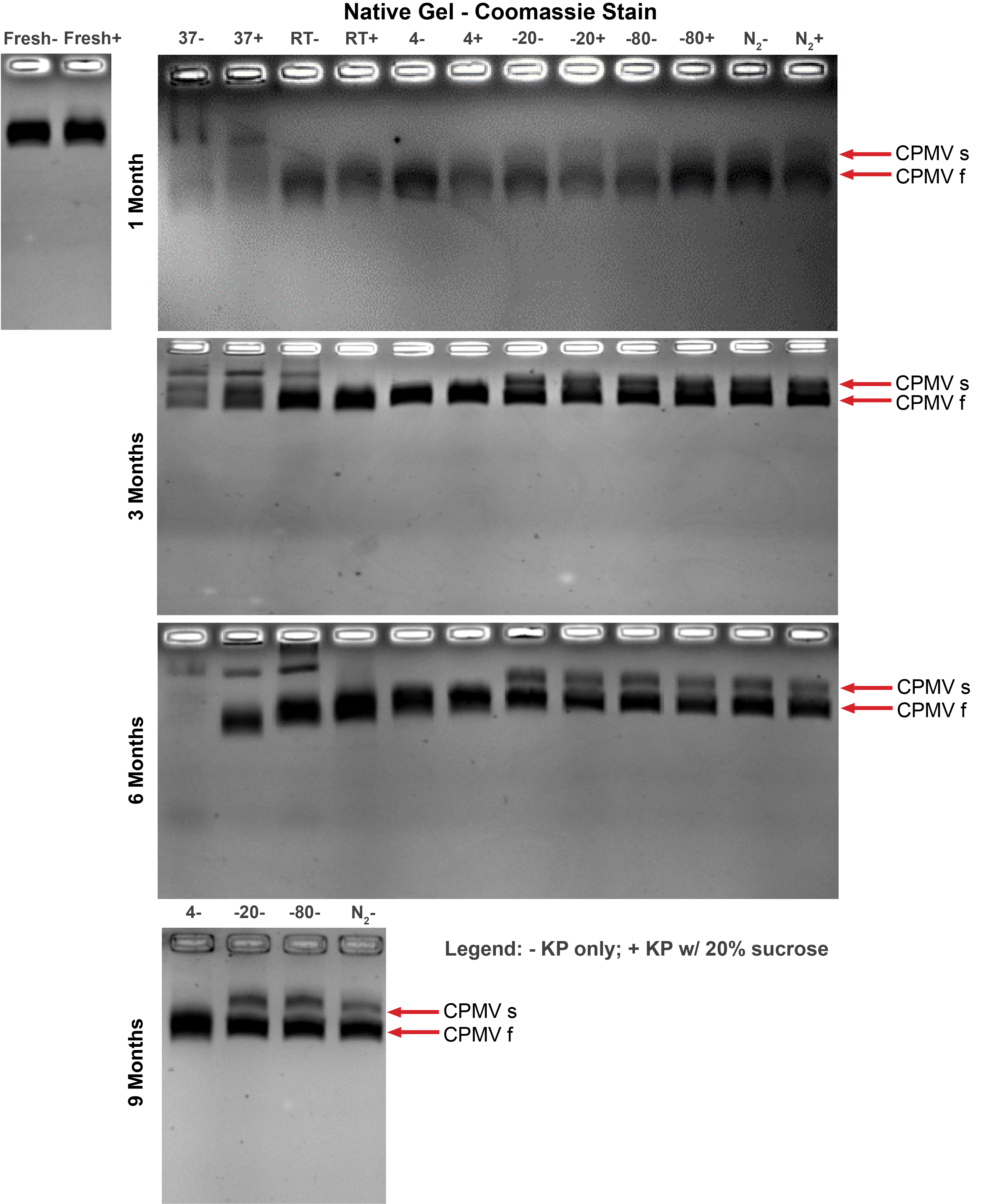


**Figure S4. Native agarose gel electrophoresis.** CPMV particles were stained with Coomassie Brilliant Blue and imaged under white light. A gel of fresh CPMV (leftmost) is compared to particles stored for 1 month, 3 months, 6 months, and 9 months.


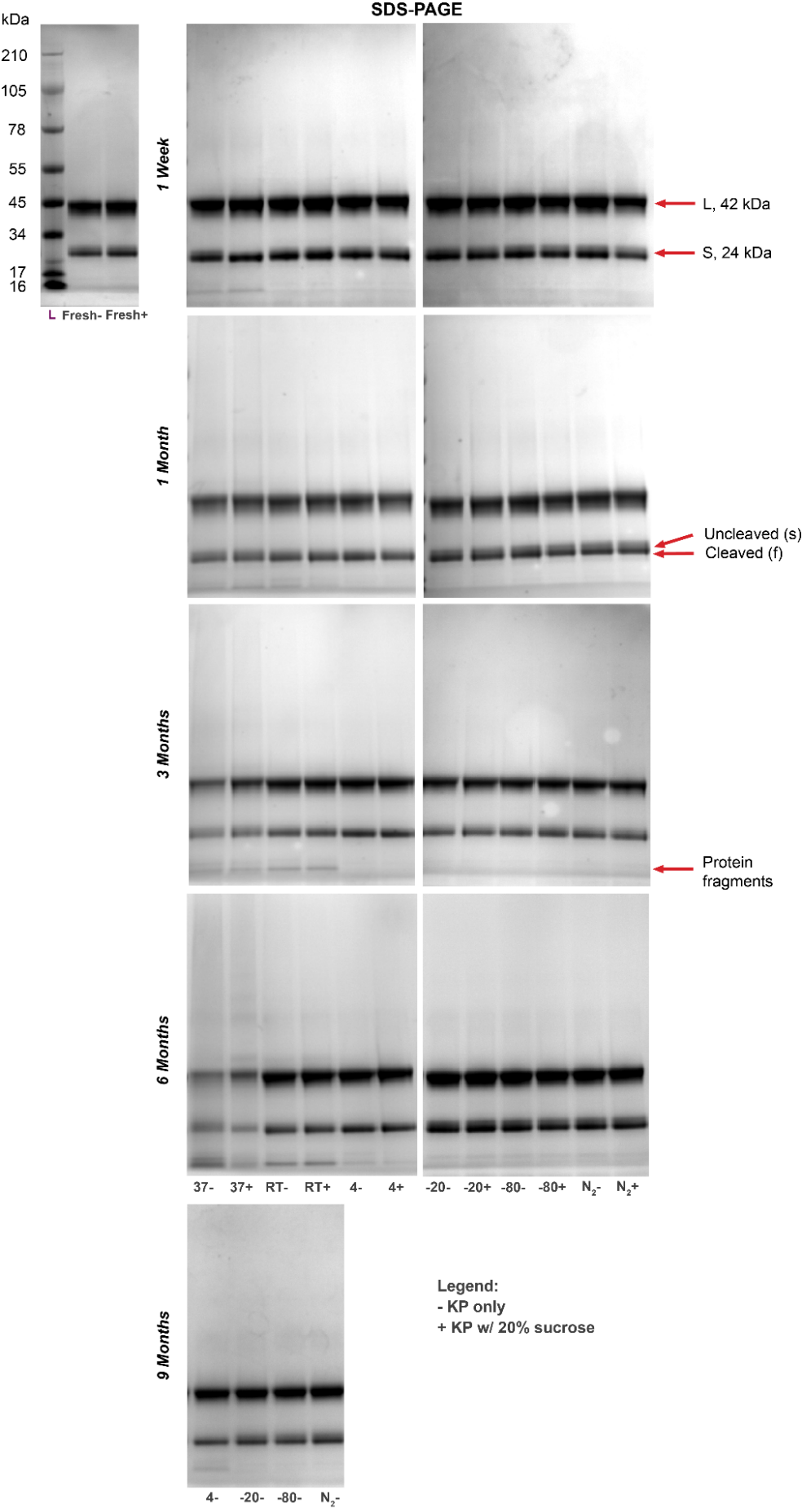


**Figure S5. SDS-PAGE.** Heat denatured CPMV particles stained with Coomassie Brilliant Blue and imaged under white light. Fresh CPMV (leftmost) is compared to particles stored for 1 week, 1 month, 3 months, 6 months, and 9 months.


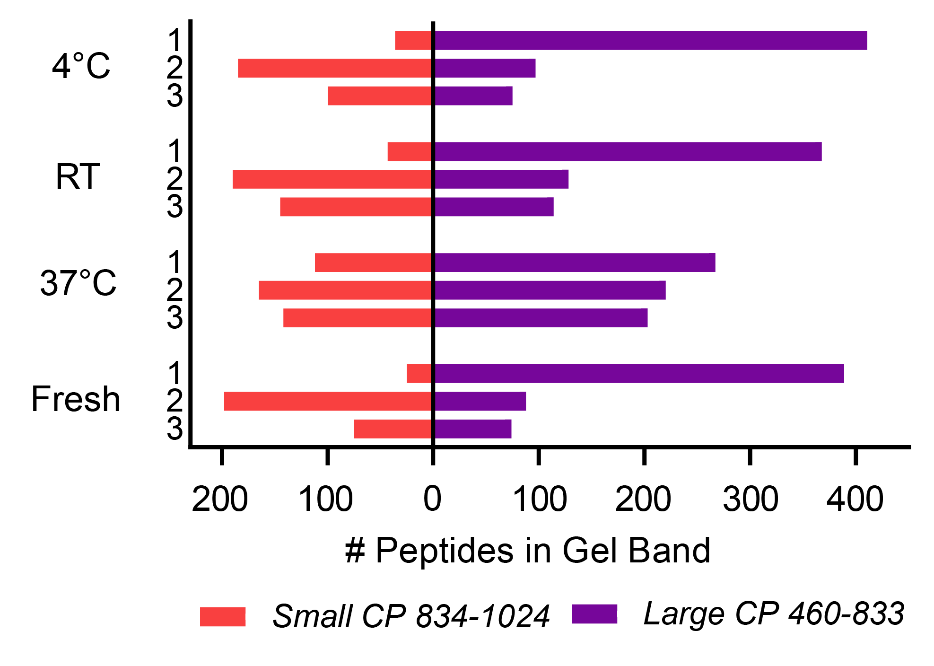


**Figure S6. LC-MS/MS.** Proteins from the (1) L, (2) S, and (3) unidentified protein fragment bands were excised from fresh, 37°C, RT, and 4°C CPMV sample SDS-PAGE gels and analyzed by LC-MS/MS to evaluate peptide content.


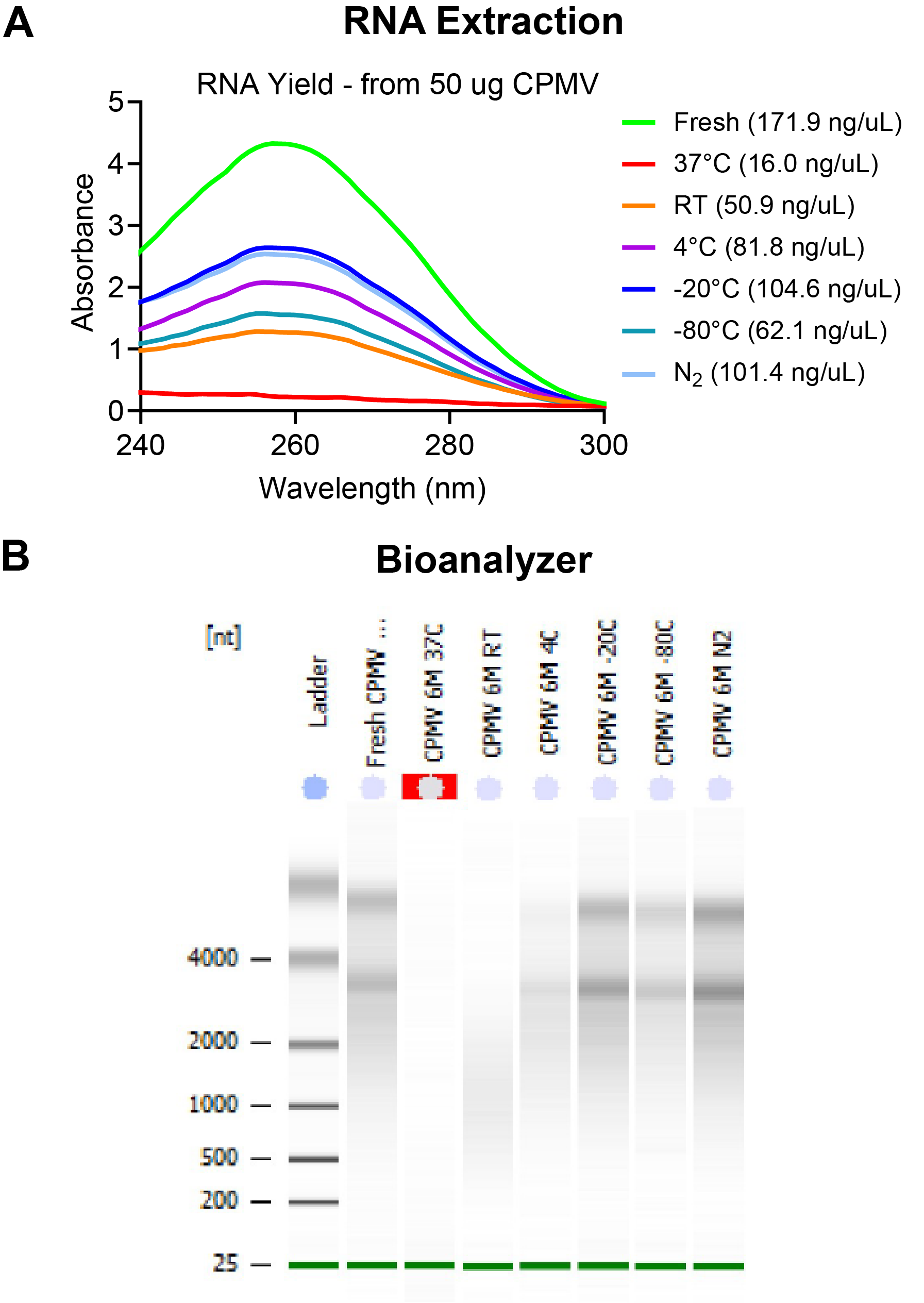


**Figure S7. RNA extracts analyzed via Bioanalyzer.** RNA was extracted from 50 µg of CPMV after 6 months in the various storage conditions then evaluated using a bioanalyzer. Minimal RNA was able to be extracted from the 37°C sample, and RNA from this condition as well as RT appeared significantly degraded. Samples stored refrigerated or colder yielded varying amounts of RNA upon extraction, but all showed characteristic RNA bands in line with fresh CPMV.


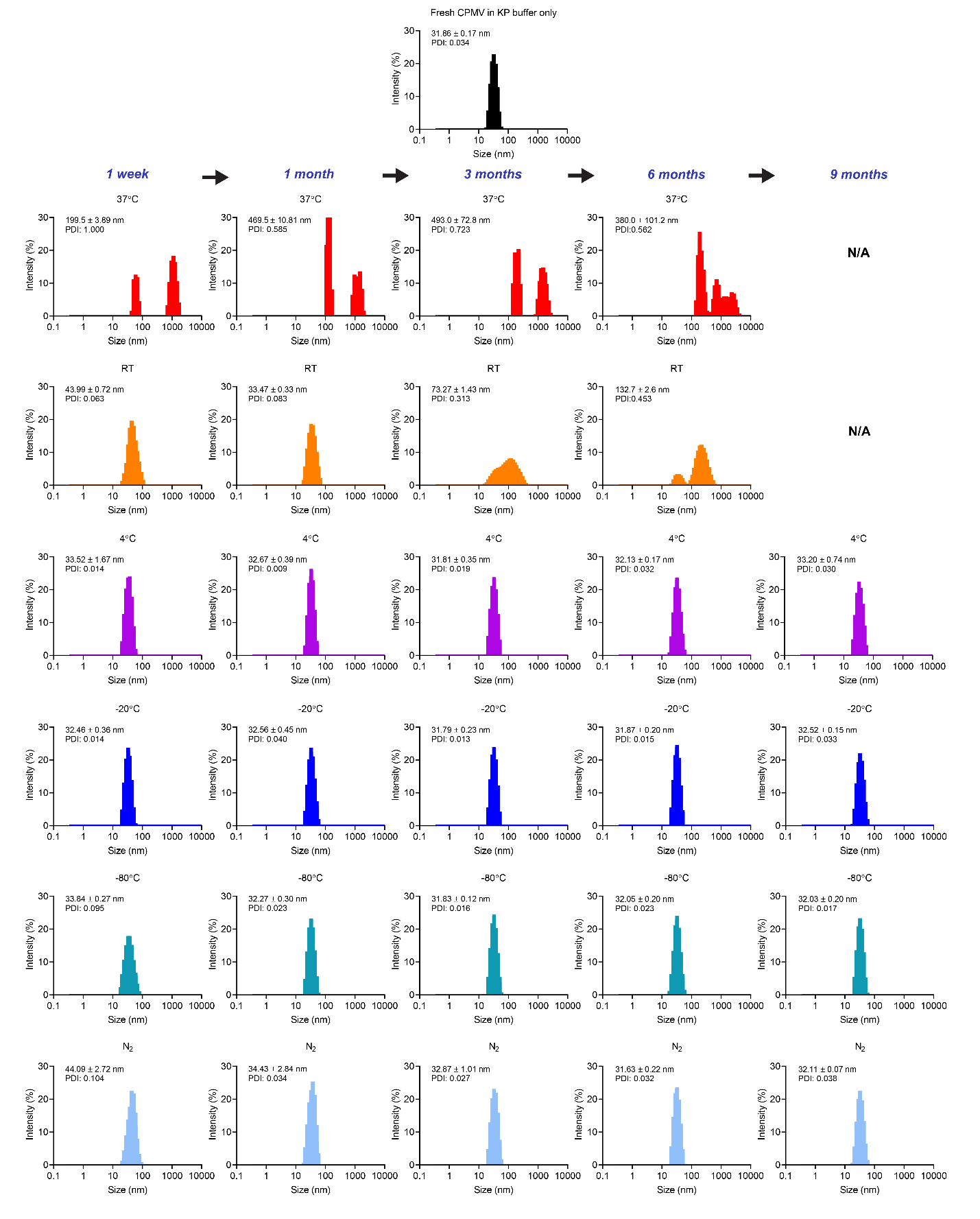


**Figure S8. Dynamic light scattering (DLS), KP buffer.** Size and degree of aggregation of CPMV were measured by DLS. Sizes are reported as the mean of 3 technical replicates ± standard deviation. Low polydispersity index (PDI) values indicate monodisperse particles and high values indicate a broad size distribution.


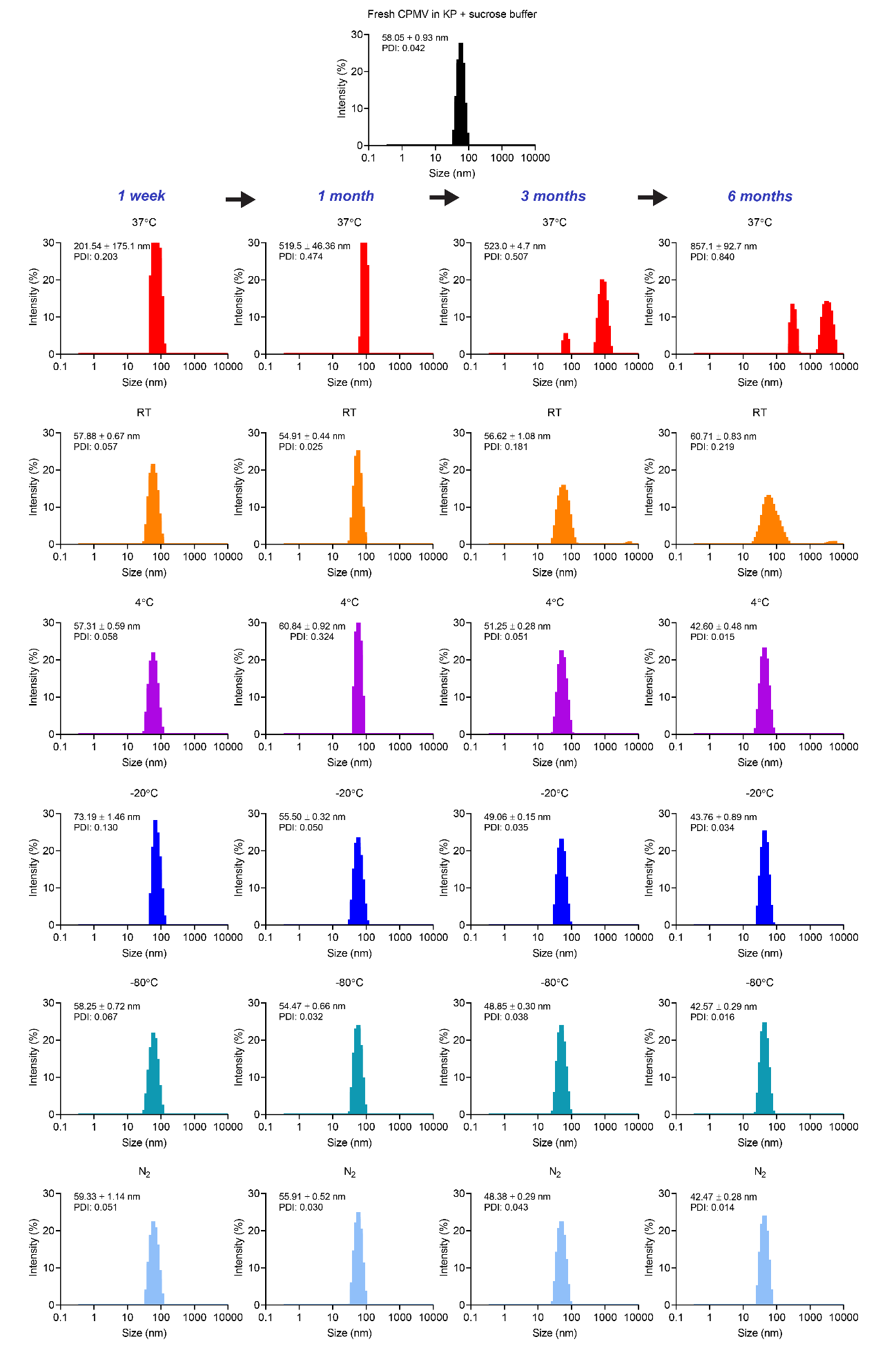


**Figure S9. Dynamic light scattering (DLS), KP buffer + 20% sucrose.** Size and degree of aggregation of CPMV were measured by DLS. Sizes are reported as the mean of 3 technical replicates ± standard deviation. Low polydispersity index (PDI) values indicate monodisperse particles and high values indicate a broad size distribution.


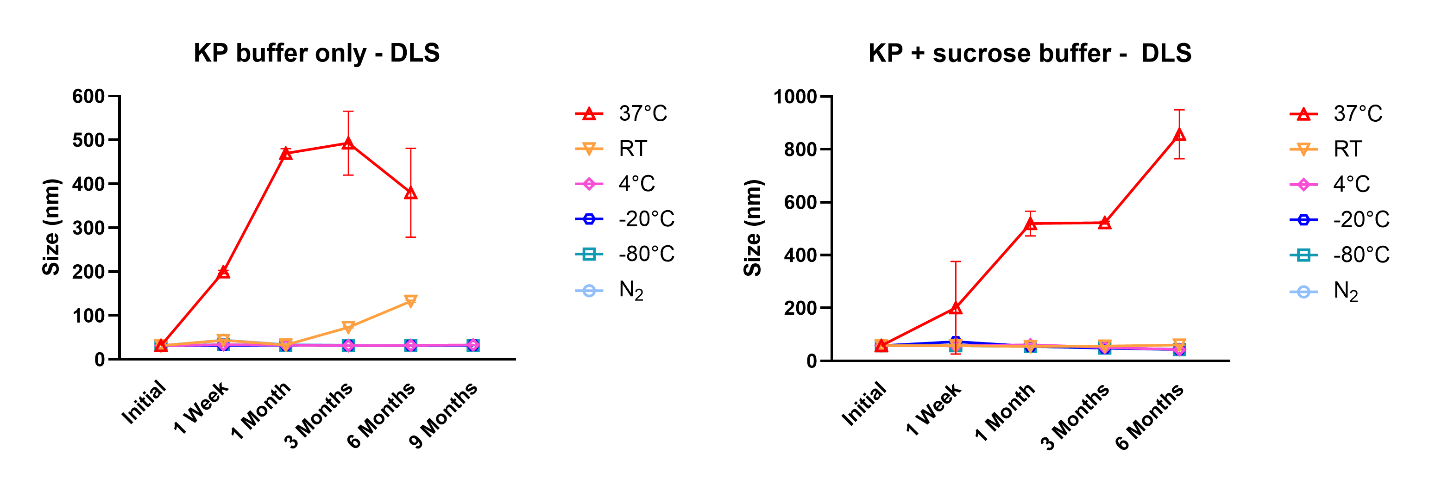


**Figure S10. Summary of DLS data, KP buffer only (left) and KP buffer + 20% sucrose (right).** The size of CPMV over time was measured by DLS. Sizes are reported as the mean of 3 technical replicates ± standard deviation.


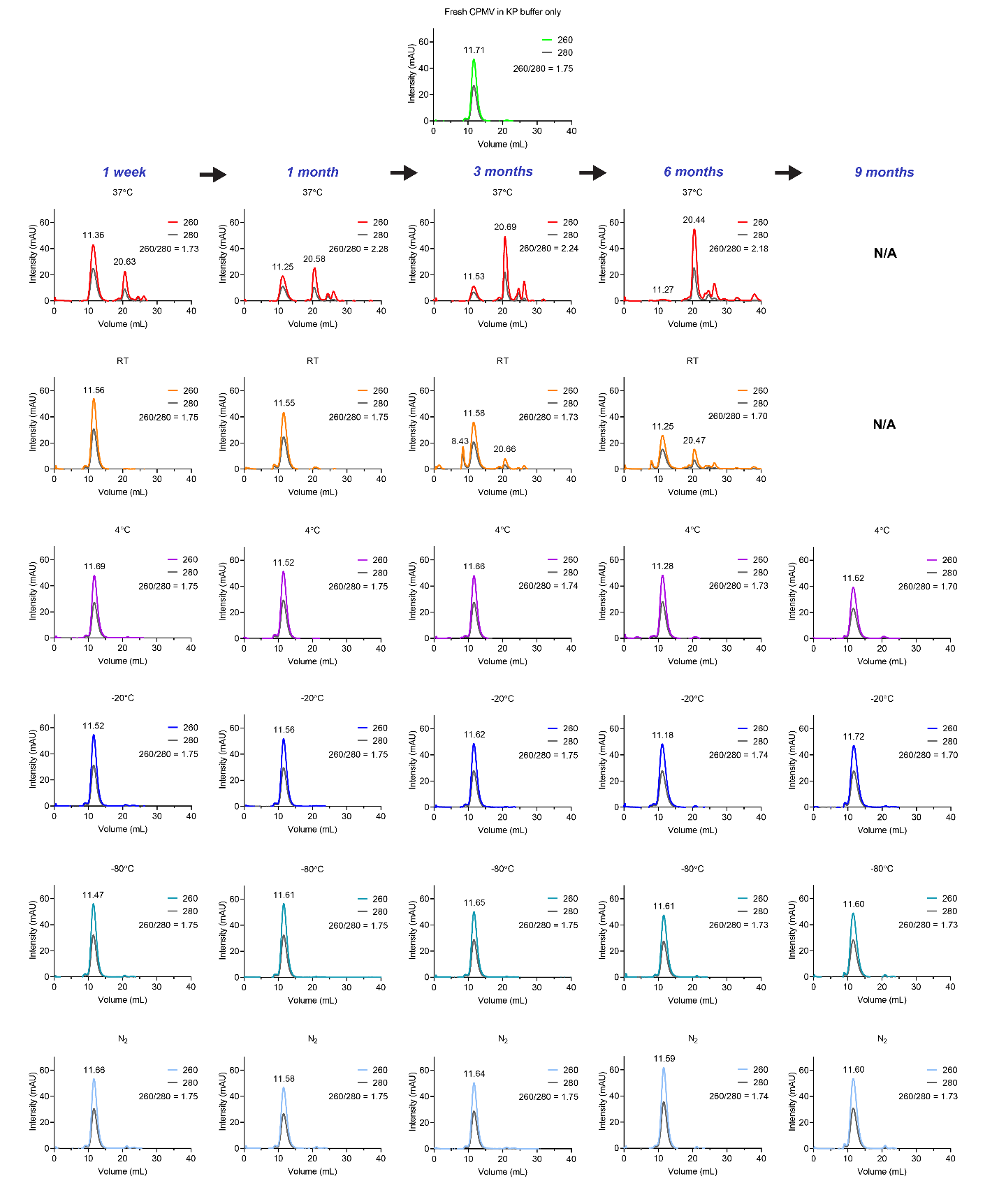


**Figure S11.** **Fast Protein Liquid Chromatography (FPLC), KP buffer.** CPMV particle size and integrity were evaluated by FPLC using a Superose 6 Increase 10/300 GL column. The characteristic elution profile for CPMV is a single peak ~11.7 mL and any peaks appearing before or after this volume are indicative of aggregation (former) or broken particles (latter). Absorbances at 260 nm (RNA) and 280 nm (protein) are expected to co-elute at a ratio (A260/280) of 1.8 for intact particles, with values ranging 1.7-1.9 deemed acceptable.


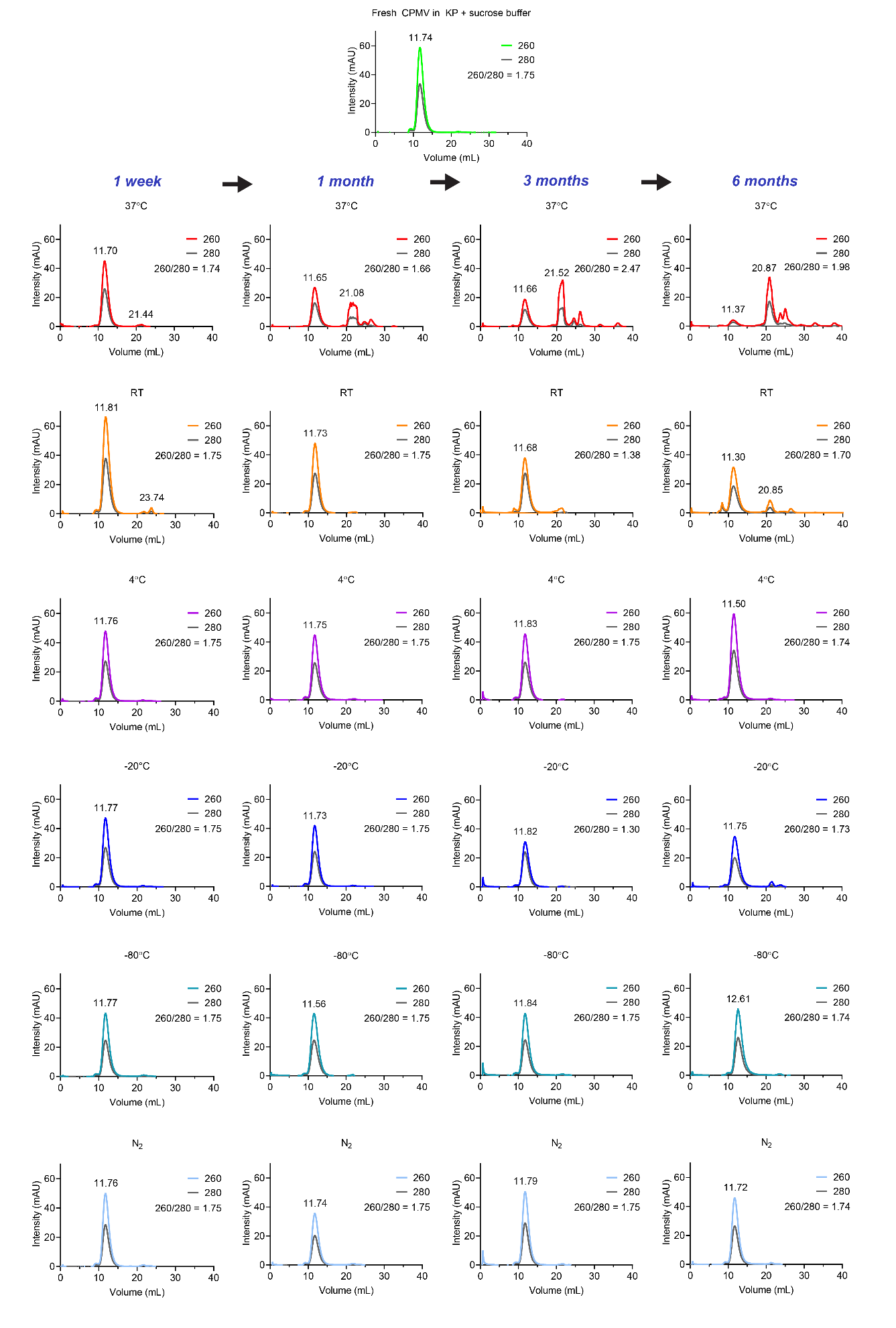


**Figure S12. Fast Protein Liquid Chromatography (FPLC), KP buffer + 20% sucrose.** CPMV particle size and integrity were evaluated by FPLC using a Superose 6 Increase 10/300 GL column. The characteristic elution profile for CPMV is a single peak ~11.7 mL and any peaks appearing before or after this volume are indicative of aggregation (former) or broken particles (latter). Absorbances at 260 nm (RNA) and 280 nm (protein) are expected to co-elute at a ratio (A260/280) of 1.8 for intact particles, with values ranging 1.7-1.9 deemed acceptable.


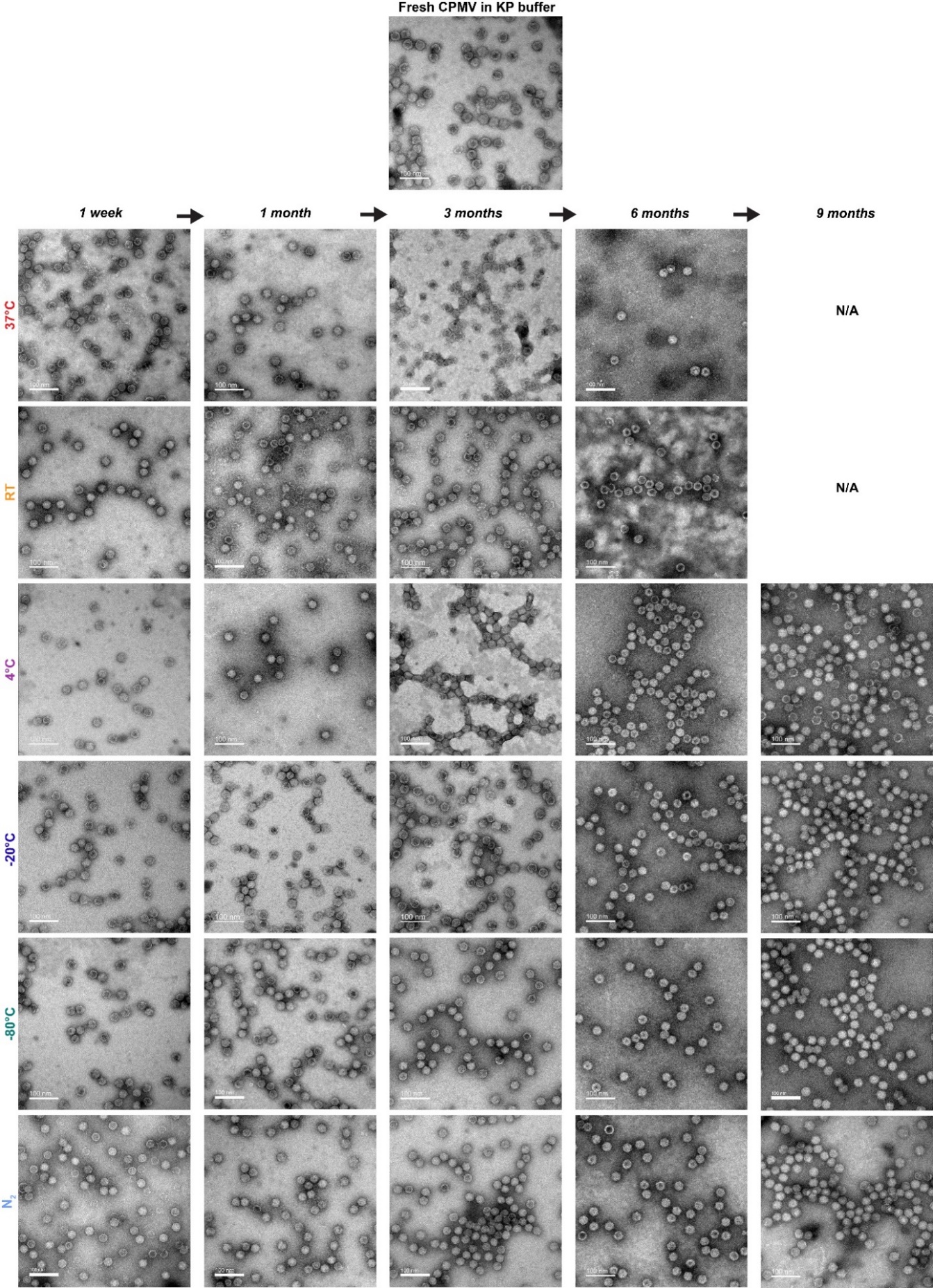


**Figure S13. Transmission Electron Microscopy (TEM), KP buffer.** Negatively stained CPMV particles were imaged via TEM at 80,000x magnification to confirm size and to inspect for visual cues of degradation. The scale bar is 100 nm.


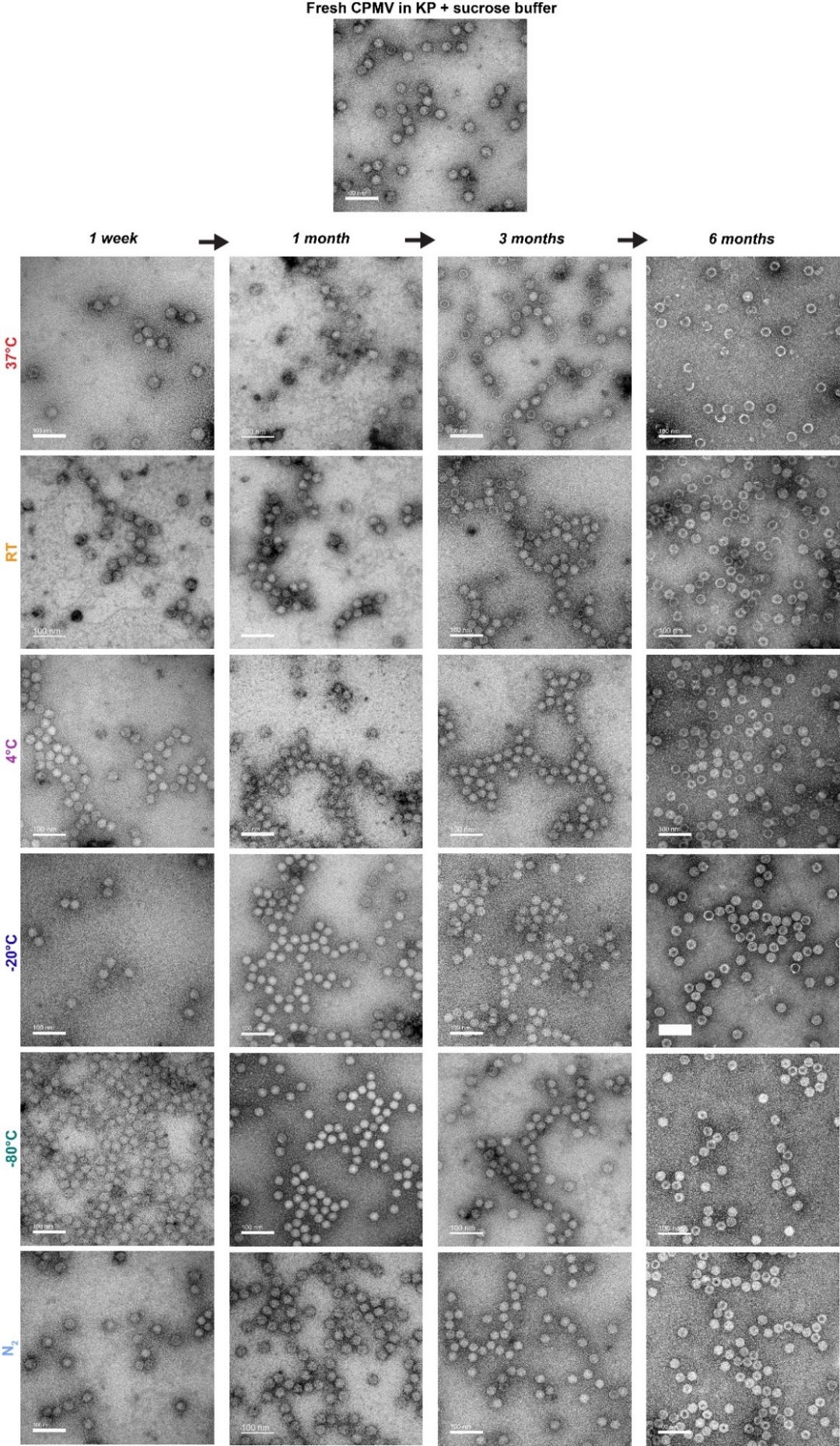


**Figure S14. Transmission Electron Microscopy (TEM), KP buffer + 20% sucrose.** Negatively stained CPMV particles were imaged via TEM at 80,000x magnification to confirm size and to inspect for visual cues of degradation. The scale bar is 100 nm.

***
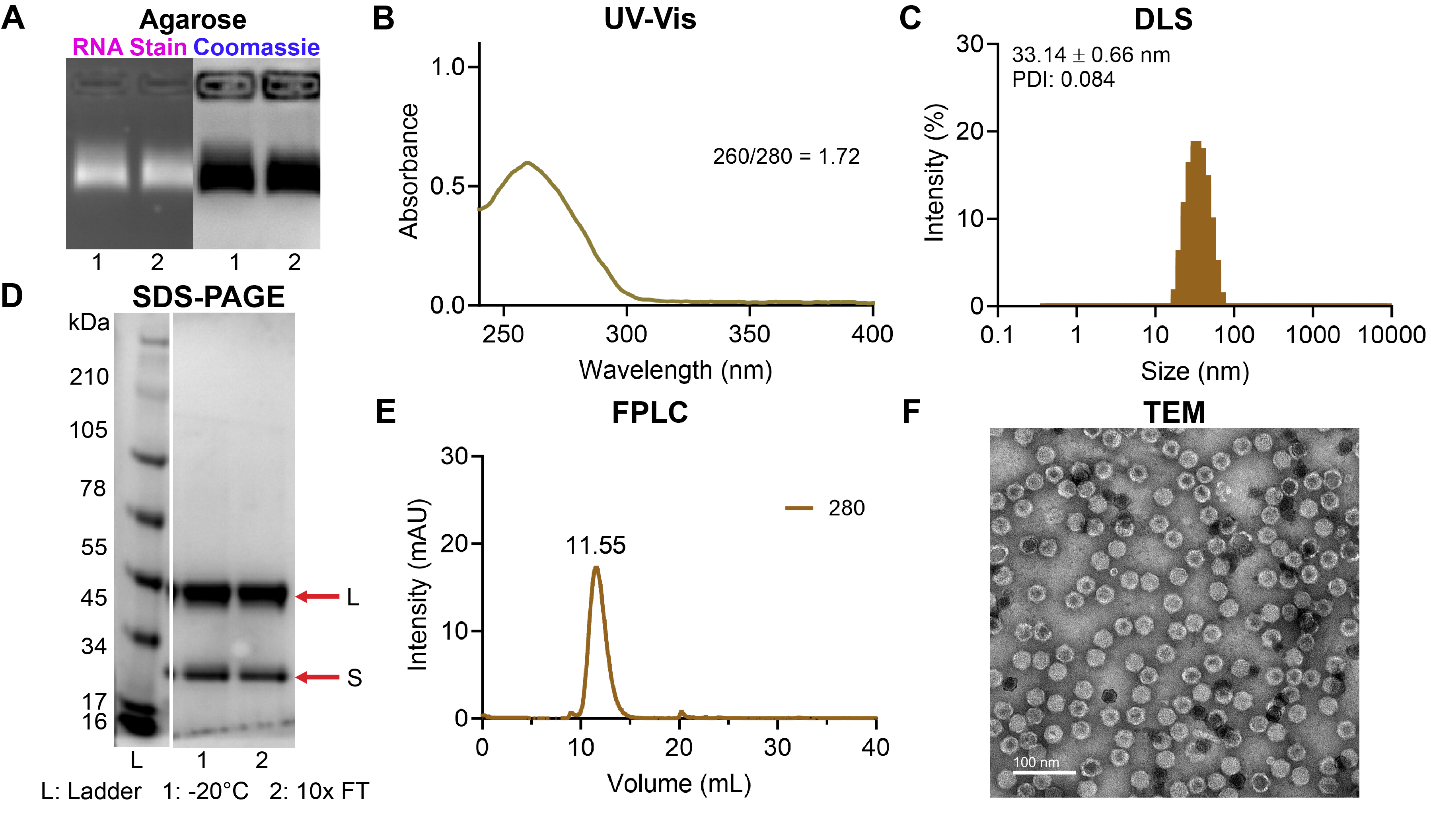
***

**Figure S15. CPMV was stored in -20°C for 3 months then subjected to 10 cycles of 24 hours each of thawing then re-freezing.** Samples were then characterized against a -20°C sample of the same age in KP buffer only that was not subjected to freeze-thaw. (A) Agarose gel electrophoresis stained with GelRed nucleic acid stain (left) and Coomassie Brilliant Blue (right) (B) UV-Vis (C) DLS (D) SDS-PAGE of heat denatured CPMV stained with Coomassie Brilliant Blue (E) FPLC (F) TEM.


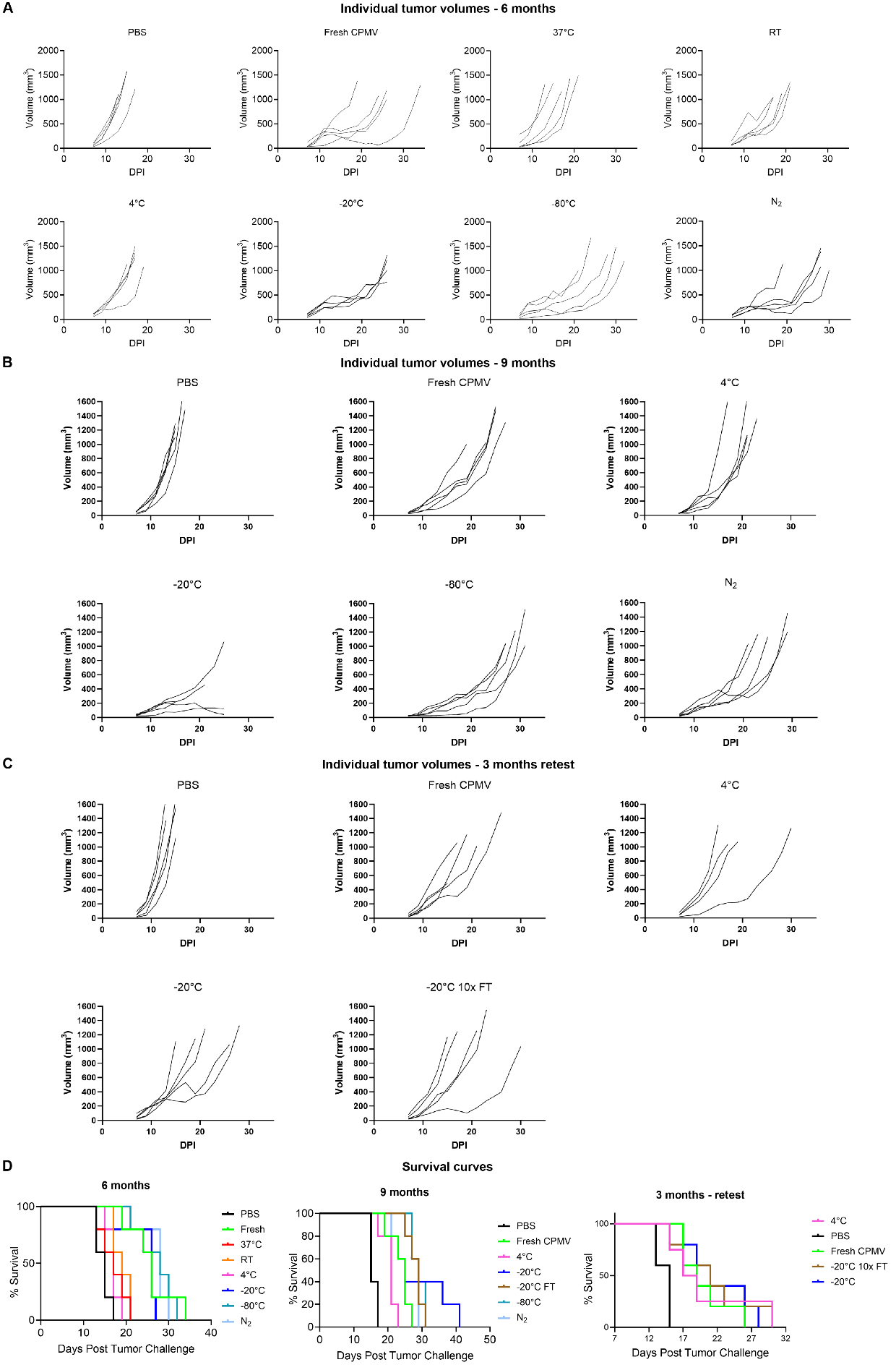


**Figure S16. Intratumoral treatment against B16F10 murine melanoma using fresh CPMV vs. aged CPMV stored in multiple conditions.** (A) Individual tumor volume growth after treatment with CPMV stored for 6 months. (B) Individual tumor volume growth after treatment with CPMV stored for 9 months. (C) Individual tumor volume growth after treatment with a new batch of CPMV stored for 3 months. (D) Survival curves for the mice in panels A-C.


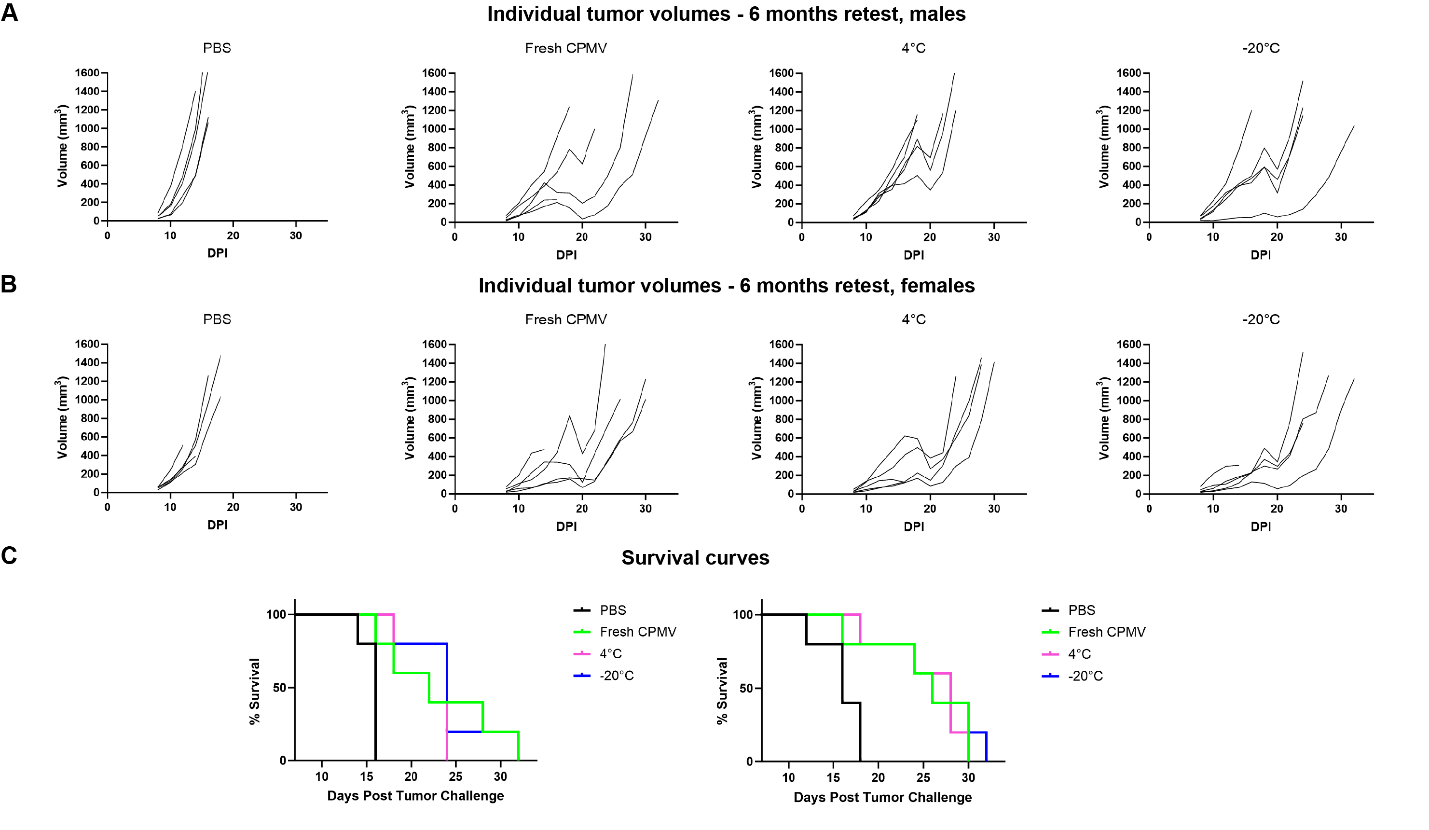


**Figure S17. Intratumoral treatment against B16F10 murine melanoma using fresh CPMV vs. aged CPMV stored in multiple conditions.** (A) Individual tumor volume growth after treatment with the new batch of CPMV stored for 6 months, male mice. (B) Individual tumor volume growth after treatment with the new batch of CPMV stored for 6 months, female mice. (C) Survival curves for the mice shown in panels A and B.


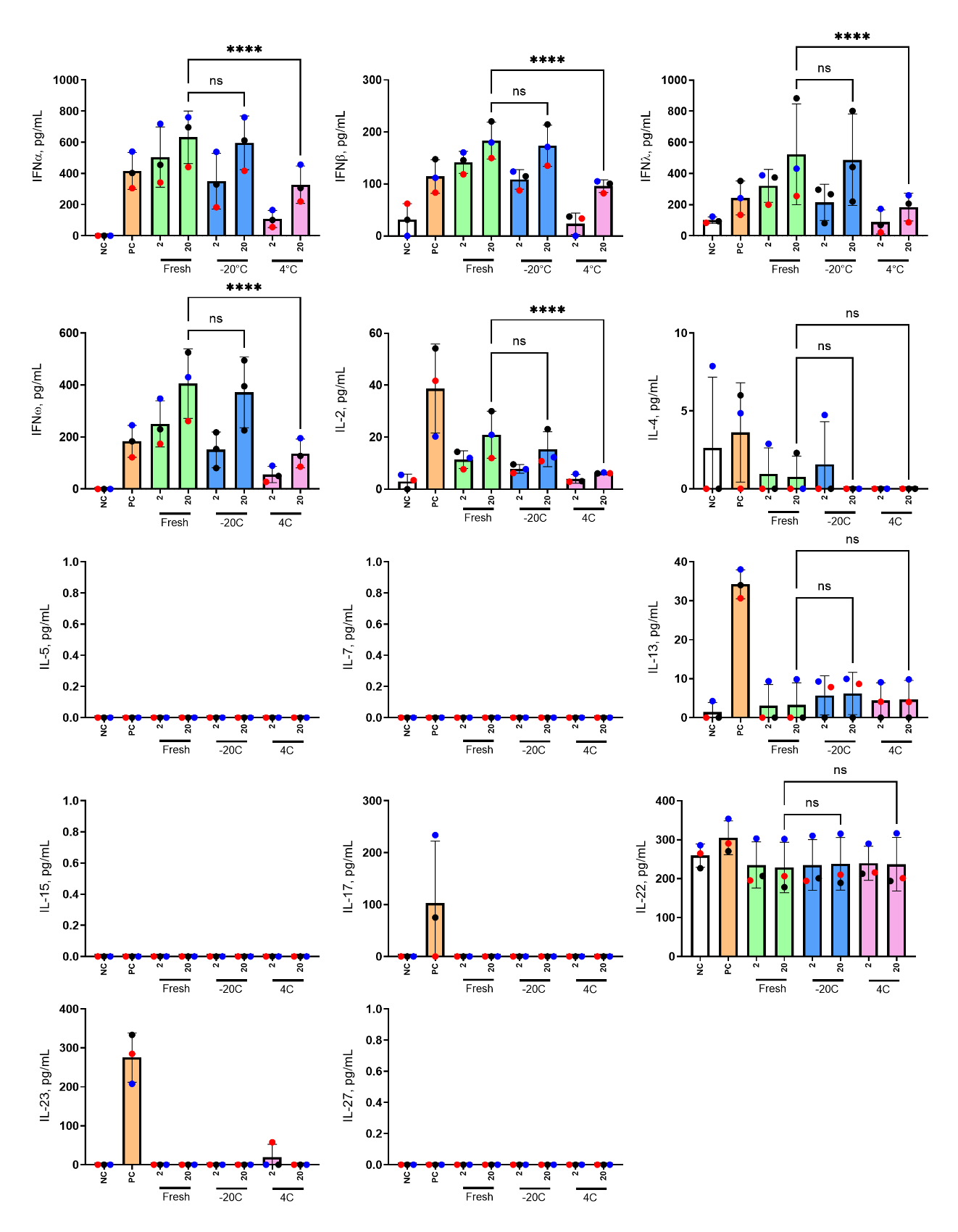


**Figure S18. Cytokine induction by fresh vs. aged CPMV.** PBMC from healthy human donor volunteers were exposed to controls or CPMV for 24 hours, and the activation of cytokine responses was assessed using a 14-plex panel. Shown here are the individual graphs of all 14 cytokine responses. Each bar shows the mean response and standard deviation of data obtained from three healthy donors. Each dot shows the mean responses (N=3) of cells from individual donors as follows: black – Donor K2J1; red – Donor Q3G6; and blue – Donor W9M9. NC = negative control (PBS); PC = positive control (5 µg/mL ODN2216 and 10 µg/mL PHA-M). Statistical significance between fresh, -20°C, and 4°C samples was calculated using Two-Way ANOVA with **** = P < 0.0001, *** = P < 0.0002, ** = P < 0.0021, * = P < 0.0332, and ns = P < 0.1234.
